# Neuron-specific activation of necroptosis signaling in multiple sclerosis cortical grey matter

**DOI:** 10.1007/s00401-021-02274-7

**Published:** 2021-02-10

**Authors:** Carmen Picon, Anusha Jayaraman, Rachel James, Catriona Beck, Patricia Gallego, Maarten E. Witte, Jack van Horssen, Nicholas D. Mazarakis, Richard Reynolds

**Affiliations:** 1 Division of Neuroscience, Department of Brain Sciences, Faculty of Medicine, Hammersmith Hospital Campus, Imperial College London, London, W12 0NN UK; 2 Centre for Molecular Neuropathology, Lee Kong Chian School of Medicine, Nanyang Technological University, Singapore, Singapore; 3 Department of Pathology, MS Center Amsterdam, Amsterdam Neuroscience, Amsterdam UMC, Amsterdam, The Netherlands; 4 Department of Molecular Cell Biology and Immunology, MS Center Amsterdam, Amsterdam Neuroscience, Amsterdam UMC, Amsterdam, The Netherlands

**Keywords:** Neurodegeneration, Necroptosis, Apoptosis, Cytokines, Meninges

## Abstract

Sustained exposure to pro-inflammatory cytokines in the leptomeninges is thought to play a major role in the pathogenetic mechanisms leading to cortical pathology in multiple sclerosis (MS). Although the molecular mechanisms underlying neurodegeneration in the grey matter remain unclear, several lines of evidence suggest a prominent role for tumour necrosis factor (TNF). Using cortical grey matter tissue blocks from post-mortem brains from 28 secondary progressive MS subjects and ten non-neurological controls, we describe an increase in expression of multiple steps in the TNF/TNF receptor 1 signaling pathway leading to necroptosis, including the key proteins TNFR1, FADD, RIPK1, RIPK3 and MLKL. Activation of this pathway was indicated by the phosphorylation of RIPK3 and MLKL and the formation of protein oligomers characteristic of necrosomes. In contrast, caspase-8 dependent apoptotic signaling was decreased. Upregulation of necroptotic signaling occurred predominantly in macroneurons in cortical layers II–III, with little expression in other cell types. The presence of activated necroptotic proteins in neurons was increased in MS cases with prominent meningeal inflammation, with a 30-fold increase in phosphoMLKL+ neurons in layers I–III. The density of phosphoMLKL+ neurons correlated inversely with age at death, age at progression and disease duration. In vivo induction of chronically elevated TNF and INFγ levels in the CSF in a rat model via lentiviral transduction in the meninges, triggered inflammation and neurodegeneration in the underlying cortical grey matter that was associated with increased neuronal expression of TNFR1 and activated necroptotic signaling proteins. Exposure of cultured primary rat cortical neurons to TNF induced necroptosis when apoptosis was inhibited. Our data suggest that neurons in the MS cortex are dying via TNF/TNFR1 stimulated necroptosis rather than apoptosis, possibly initiated in part by chronic meningeal inflammation. Neuronal necroptosis represents a pathogenetic mechanism that is amenable to therapeutic intervention at several points in the signaling pathway.

**Supplementary Information:**

The online version contains supplementary material available at 10.1007/s00401-021-02274-7.

## Introduction

The progressive stages of multiple sclerosis (MS) are characterised pathologically by the accumulation of chronic demyelinated lesions in the white matter (WM) [31], variable axon damage and loss in the WM [26], diffuse changes in normal-appearing WM [17, 27] and increasing cortical grey matter (GM) pathology [3, 18, 27, 45]. The degree of GM pathology correlates better with the rate of clinical progression, physical disability and cognitive impairment than the WM lesion burden on MRI [4, 11, 16, 37] and neurodegeneration in the GM is now thought to be one of the main drivers of this accumulating irreversible clinical disability. Neuronal loss, axon and neurite damage, and synaptic dysfunction and loss, all contribute to this GM neurodegenerative pathology [14, 23, 35, 36, 45, 58]. However, the mechanisms underlying cortical neurodegeneration and subsequent GM atrophy are still unclear, although it is suggested to be stimulated by a cascade of events triggered by chronic inflammation [3, 12]. The extent of neuronal loss in the cortical GM associates with compartmentalized inflammation within the leptomeninges [14, 18, 35], which is suggested to drive the underlying pathology, possibly by the diffusion of pro-inflammatory cytokines into the GM [21]. The pro-inflammatory cytokine, tumour necrosis factor (TNF), which is known to stimulate cell death in a wide variety of non-CNS chronic inflammatory conditions, is elevated in active lesions, CSF and meninges of MS patients, and is associated with increased GM pathology [34, 50, 53], making it an obvious candidate for further study.

Transcriptomics analysis of cortical GM suggested a dysregulation of TNF signaling towards the activation of necroptosis in the presence of increased meningeal inflammation [33]. While binding of soluble TNF (sTNF) to the TNF receptor 1 (TNFR1) can regulate both pro-survival and cell death pathways, binding of transmembrane TNF (tmTNF) to the TNF receptor 2 (TNFR2) mediates mainly cell survival and tissue regeneration [1, 32, 46, 57, 61]. Death signaling following sTNF/TNFR1 interaction is initiated when receptor-interacting protein kinase 1 (RIPK1) is deubiquitinated, leading to the activation of caspase-8 that leads to apoptosis [40, 41, 43, 51]. However, when caspase-8 is inhibited, RIPK1 interacts with receptor-interacting protein kinase 3 (RIPK3), inducing autophosphorylation [24, 56], recruitment and phosphorylation of mixed lineage pseudokinase ligand (MLKL), leading to the formation of the necrosome [28, 55]. MLKL oligomers translocate to the plasma membrane and execute plasma membrane rupture [5, 7].

These observations led us to hypothesize that TNF produced in the meninges diffuses into the underlying grey matter to cause TNFR1-dependent necroptotic neuronal cell death, either directly, or by activating glial cells. Here we report an upregulation in multiple stages of TNFR1 signaling towards necroptosis activation in cortical neurons in the progressive MS brain, indicating that neurodegeneration is occurring via necroptosis rather than apoptosis. In addition, chronically increased levels of TNF in the CSF in an in vivo model reproduced our findings in MS tissue and were associated with neurodegeneration and necroptosis activation in cortical neurons. These findings reveal an inflammatory mechanism of cell death in cortical neurons that might result in new potential therapeutic avenues to target neurodegeneration in progressive MS.

## Materials and methods

### Tissue samples

The UK MS Society Tissue Bank at Imperial College London provided all post-mortem tissue for this study. Fully informed consent was obtained for post-mortem donation under ethical approval by the National Research Ethics Committee (08/MRE09/31). The demographic data and clinical and neuropathological features of the SPMS cases and controls are shown in suppl. Table 1. Neuropathological analysis and patient history confirmed MS diagnosis as previously reported [49]. For the analysis of mRNA and protein levels, 1 cm × 2 cm × 2 cm snap-frozen blocks were obtained from the cingulate gyrus, precentral gyrus, insula and temporal gyrus from 28 SPMS (median post-mortem delay (PMD) = 17 h, range: 8–27; median age at death = 53 years, range: 38–70) and ten control brains (median PMD = 21 h, range: 10–48; median age at death = 63 years, range: 35–82). For the quantitative immunohistochemical analysis, 6 μm thick, formalin‐fixed, paraffin‐embedded sections from 17 SPMS (median PMD = 18 h, range 8–27; median age at death = 54 years, range: 38–27) cases and 11 controls (median PMD = 21 h, range: 10–48; median age at death = 63 years, range: 35–82) were cut from the superior frontal cortex and cingulate gyrus, based on tissue availability.

### Immunohistochemistry

Individual antibody details are listed in suppl. Table 2. Human paraffin-embedded sections were deparaffinised, rehydrated and subjected to epitope retrieval. Sections were blocked with 10% normal horse serum (NHS) followed by overnight incubation with primary antibody and then incubated with ImmPRESS HRP-conjugated secondary antibodies (Vector Laboratories). Slides were visualized with ImmPACT-DAB (Vector Laboratories) as the chromogen. Sections were counterstained with haematoxylin and DePex mounted. Dual colour IHC was performed sequentially. After detection of the primary antibody with ImmPACT-DAB, incubation with the second primary antibody was conducted and detected using the ABC-alkaline phosphatase detection system, using Vector Blue as substrate. When using snap-frozen tissue for IHC, slides were pre-treated with cold methanol and then followed the same steps as for paraffin tissue.

For immunofluorescence, sections were fixed with 100% methanol at − 20 °C, blocked and incubated overnight with primary antibodies. Sections were incubated with the appropriate secondary antibody conjugated to a fluorochrome, as described previously [35] and nuclei counterstained with DAPI (Sigma-Aldrich) and mounted with Vectashield Antifade Mounting Media (Vector Laboratories).

### Protein extraction

Grey matter samples from MS and control tissue blocks were dissected carefully on a Leica cryostat, homogenized in RIPA buffer (Thermo Scientific) containing protease and phosphatase inhibitors (Thermo Scientific), and incubated on ice for 20 min at 4 °C. The protein extract was centrifuged at 120,000*g* for 15 min at 4 °C. The resulting supernatant was taken as the RIPA soluble fraction. Pellets were washed in TBS and homogenized in 6 M urea/5% SDS for 30 min at room temperature (RT). The samples were stored at − 80 °C until used for Western blotting. To extract proteins in native condition, tissue was homogenised in TBS containing 0.1% of Triton X-100, incubated for 10 min at RT and centrifuged at 16,000*g* for 10 min. The cytoplasmic and nuclear fraction was extracted using the NE-PER™ Nuclear and Cytoplasmic Extraction Reagents (Thermo Scientific) according to the manufacturer’s guidelines.

### Western blotting

Protein concentration was measured using a Pierce BCA protein assay kit (Thermo Scientific). Subsequently, 10–50 μg of protein was loaded onto 4–12% Bis–Tris gels (Thermo Scientific) and transferred to polyvinylidene difluoride (PVDF) membranes for 1.30 h. The membranes were incubated in 5% BSA or milk for 1 h at room temperature and incubated overnight at 4 °C with the appropriate primary antibodies. Individual antibodies used are listed in suppl. Table 2. The next day, the blots were washed three times with TBS-T for 10 min and incubated with the specific secondary antibodies (1:20,000, Jackson laboratory) for 1 h at RT. The blots were then washed with TBS-T, and imaged/quantified using a Syngene G:Box. For the detection of MLKL monomers and dimers, proteins were run on 3–8% Tris–acetate gels (Thermo Scientific) in non-reducing conditions.

### RNA extraction and RT-PCR

For RNA extraction, grey matter regions in each brain tissue block were dissected from serial sections as for protein extraction. The grey matter tissue samples were then homogenized and processed for total RNA extraction using PureLinkTM RNA Mini kit (Life Technologies Corporation) as per the manufacturer’s protocol. Purified total RNA (100 ng) was used from each sample for One-step real-time reverse transcriptase quantitative polymerase chain reaction (RT-qPCR) using the iTaqTM Universal SYBR^®^ Green One-Step kit (Bio-Rad) in the StepOnePlusTM Real-Time PCR system (Applied Biosystems). The primers used for *mlkl*, *ripk3*, *tnfr1*, *ripk1* and *xpnpep1* were the commercially available PrimePCRTM SYBR^®^ Green assay primers (Biorad). For each sample, reactions were set up in triplicate with the following cycling protocol: 50 °C for 10 min, 95 °C for 1 min, 40 cycles with a 3-step protocol (95 °C for 15 s, 60 °C for 1 min), and a final melting curve analysis with a ramp from 65 to 95 °C. Relative quantification of mRNA levels from various treated samples was determined by the ∆∆Ct method [29], after normalizing with the corresponding *xpnpep1* levels [8] from the samples.

### Immunoprecipitation

Samples from MS and control brain grey matter were homogenized in lysis buffer (EDTA 1 mm, EGTA 1 mm, 0.1%, Triton X-100, 2 mm MgCl_2_, 150 mm NaCl, 50 mm pH 7.5 Tris–HCL) containing protease and phosphatase inhibitors. 50 μg of protein per sample was incubated with 6 μg of anti-RIPK3 (R&D) and 50 μl of Dynabeads Protein G (Thermo Scientific) while rotating overnight at 4 °C. The beads were then washed three times with lysis buffer and mixed with loading NuPAGE LDS sample buffer (Thermo Scientific). The beads were boiled at 70 °C for 10 min and placed on ice. Samples were loaded onto a 10% Bis–Tris gel (Thermo Scientific). Gels were transferred to a PVDF membrane and incubated overnight at 4 °C with anti-MLKL followed by 1 h incubation at 25 °C with anti-rabbit IgG-HRP secondary antibody. Blots were then washed and developed with Clarity (BioRad).

### Animals

Eight- to ten-week-old female Dark Agouti rats (140–160 g; total *n* = 9) were obtained from Janvier Labs (France) and maintained in groups of four in a 12 h light/dark cycle and had ad libitum access to food and water. The UK Home Office approved all procedures.

### Lentiviral vector production

Lentiviral vectors carrying the human TNF and IFNγ genes were produced as described elsewhere [21]. Briefly, 293T cells were transiently transfected with the HIV-1 vector plasmid pRRLsincppt-CMV-TNF-WPRE or pRRLsincppt-CMV-IFNƴ -WPRE), the packing vector containing HIV-1 gag/pol gene (pMD2-LgRRE), HIV-1 Rev (pRSV-Rev) and VSVG envelop plasmids using calcium phosphate. After 16 h the medium was replaced with fresh medium supplemented with 10 mM sodium butyrate. At 36 h, vector-containing medium was harvested and filtered through a 0.45-μm filter. The supernatant was then centrifuged overnight. The pellet was concentrated by ultracentrifugation and resuspended with TSSM (10 mM Tromethamine, 100 mM NaCl, 10 mg/ml sucrose and 10 mg/ml mannitol) over several hours. The genome copy number was calculated using the Clontech Lenti-X qRT-PCR Titration kit (Takara).

### Intracerebral injection of lentiviral vectors

Surgeries were performed as previously reported [13]. In brief, rats were anaesthetized with isofluorane and a 2 mm hole was drilled in the midline 0.9 mm caudal to bregma. A finely calibrated glass capillary needle attached to a Hamilton 10 μl syringe was then inserted stereotactically to a depth of 2.3 mm below the dural surface, down the sagittal sulcus. The rats were then injected with 4 µl of a lentiviral mixture (5 × 10^8^ genomic copies (GCs) of TNF and 5 × 10^7^ GCs of INFγ) a rate of 0.20 μl/ml. The needle was left in place for 5 min to allow diffusion of the sample from the area and then slowly withdrawn. Animals were perfused after 28 days with 4% paraformaldehyde in PBS (PFA) under sodium pentobarbitone anaesthesia. The brains were removed, further fixed overnight in 4% PFA and then cryoprotected in 30% sucrose in PBS before embedding in OCT and immersion in isopentane cooled on dry ice.

### Primary cortical neuron culture

Primary cortical neurons were obtained from P1 Sprague–Dawley rat pups. Cerebral cortices were dissected in ice-cold HBSS –Ca^2+^/–Mg^2+^ (plus 10 mM HEPES, pH 7.3), treated with 0.25% papain, washed in plating medium and incubated for 1 h at 37 °C, pipetting up and down every 15 min until no remaining clumps were observed. The resulting cells were seeded at 1 × 10^5^ cells/well on Poly-d-lysine-coated 96 well plates (10 mg/ml, Sigma-Aldrich) in media containing 10% heat-inactivated FBS (Invitrogen), DMEM (Invitrogen) and penicillin/streptomycin (Invitrogen). After 2 h, the plating media was changed to Neurobasal™ Plus medium (Invitrogen) supplemented with 2% B27 Plus (Invitrogen), Glutamax (Invitrogen) and penicillin/streptomycin (Invitrogen). The culture was maintained in a humidified atmosphere of 5% CO_2_ in air at 37 °C. All experiments were performed at 10–12 days in vitro (10–12 DIV) at which point the purity of the cultures was 98.7% NeuN+ cells (CL = 96–99% from *n* = 6 experiments). The only contaminating cells were a small number of GFAP+ astrocytes. Treatment with rat TNF (Peprotech), SMAC mimetics (Tocris) and Z-VAD-FMK (R&D Systems) was performed at the concentration indicated in the text. Necroptosis inhibitors GSK-547 (Sigma-Aldrich), GSK-872 (Sigma-Aldrich) and necrosulfonamide (Sigma-Aldrich) were added to the cultures for 24 h at the concentration indicated in the text.

### LDH assay

Cell cytotoxicity was determined by measuring the lactate dehydrogenase (LDH) release (LDH Assay kit) on primary cultured neurons. Supernatant (20 μl) was collected from the neuronal cultures under different conditions and samples were processed following the kit instructions. The LDH released was measured in a TECAN reader at 450 nm.

### Image acquisition and analysis

Immunohistochemistry slides stained for MOG, HuC/D, HLA-DR, RIPK1, pRIPK3, pMLKL and TNFR1 were digitized by whole slide scanning using an Aperio SCF400F scanner (Leica, Wetzlar, Germany). Image files were handled using QuPath v0.2.0. software to measure areas of demyelination. The entire GM fraction was traced and measured for each MOG‐stained slide before measuring the area of individual GM lesions. The mean GM lesion area was reported per section and per case as the percentage of total GM (suppl. Fig. 1). HLA-DR+ and TNFR-1 immunoreactive cells were counted manually from strips of sulcal grey matter extending from the pial surface to the WM, separating the data into subpial layer I, layers II–III and layers V–VI. HLA-DR+ cells were also counted within the subarachnoid space as a marker of leukocyte infiltration (suppl. Fig. 1). HuC/D, RIPK1, pRIPK3 and pMLKL cell densities were measured with the “Positive Cell Detection of a Region of Interest” tool in QuPath. Cortical layers II–III and V–VI were identified, and regions of interest were selected in each section. To identify only neurons, we restricted the analysis to immunostained features with an area > 60 μm^2^. To determine the levels of perivascular and meningeal inflammation, the total number of CD3+ cells/mm and CD20+ cells/mm was assessed as previously described and digitized using an Olympus BX63 microscope (suppl. Fig. 1) [18]. Three regions of the meninges were selected in the subarachnoid space and 8-perivascular space profiles were selected for each slide and cells counted manually. Cases showing more than 50 (CD3+ or CD20+) cells packed within the subarachnoid spaces were considered as the high meningeal inflammation group (high-inf) and less than 50 as the low inflammation group (low-inf). Sections from human post-mortem tissue labelled by double immunohistochemistry for necroptotic proteins were imaged with an Olympus BX63 scanning microscope. Scanning of the cortical ribbons was performed at 20 × magnification and regions of interest were selected in layers II–III and V–VI and cells co-localizing NeuN with pRIPK3, cleaved caspase 3 and pMLKL counted manually with ImageJ software.

Immunofluorescence sections from the post-mortem human tissue and rat sections were imaged with either an epifluorescence Olympus BX63 scanning microscope or a SP8 Leica confocal microscope. Neuronal numbers in rat tissue were counted using NeuN+ staining with automatic cell counting using the software QPath, as described above. Regions of interest were drawn outlining layers II and layer III and layers V and VI within the cingulate cortex. Iba-1+ and GFAP+ cells were counted manually at 20 × using QPath, in the same regions of interest as the NeuN staining. To analyse the number of NeuN+ cells co-expressing TNFR1, pRIPK3 and pMLKL, we selected regions of interest in layers II–III and V–VI and counted manually at 20 × using ImageJ software. For all the immunofluorescence analyses, the digital image settings were fixed during acquisition from each experiment. The total number of cells for all the studies is given as total cells/mm^2^.

In vitro experiments were imaged with an Olympus BX63 scanning microscope. Quantitative analysis of pixel intensity (NfH-20, NeuN, pRIP1, pMLKL) was performed with ImageJ using regions of interest at 20 ×. To minimize variability between images, pixel intensity was normalized to an unstained area and the exposure time and microscope setting were fixed throughout the acquisition. The neurite degeneration index was assessed in immunofluorescence images of 200 KDa neurofilament protein-immunostained primary cortical neurons using ImageJ. Relative neurite integrity was based on the ratio of fragmented to total neurites longer than 150 µm.

### Statistical analysis

All human post-mortem data was assumed to be sampled from a non-Gaussian distribution and non-parametric analysis methods applied. The difference between two groups was compared using the unpaired Mann–Whitney test, whilst the Kruskal–Wallis test with Dunn’s multiple comparison post-test was used when comparing three or more groups. Spearman correlation was used to test for associations between groups and the Spearman *r* and *p* values reported in each instance. To analyse in vivo and in vitro experiments we used one-way ANOVA with Bonferroni’s post-hoc correction for multiple comparisons. A two-sided *p* value < 0.05 was considered significant.

### Data availability

All the data have been made available as part of the manuscript.

## Results

### TNFR1/RIPK1 signaling is upregulated in neurons in MS grey matter

**Fig. 1:**
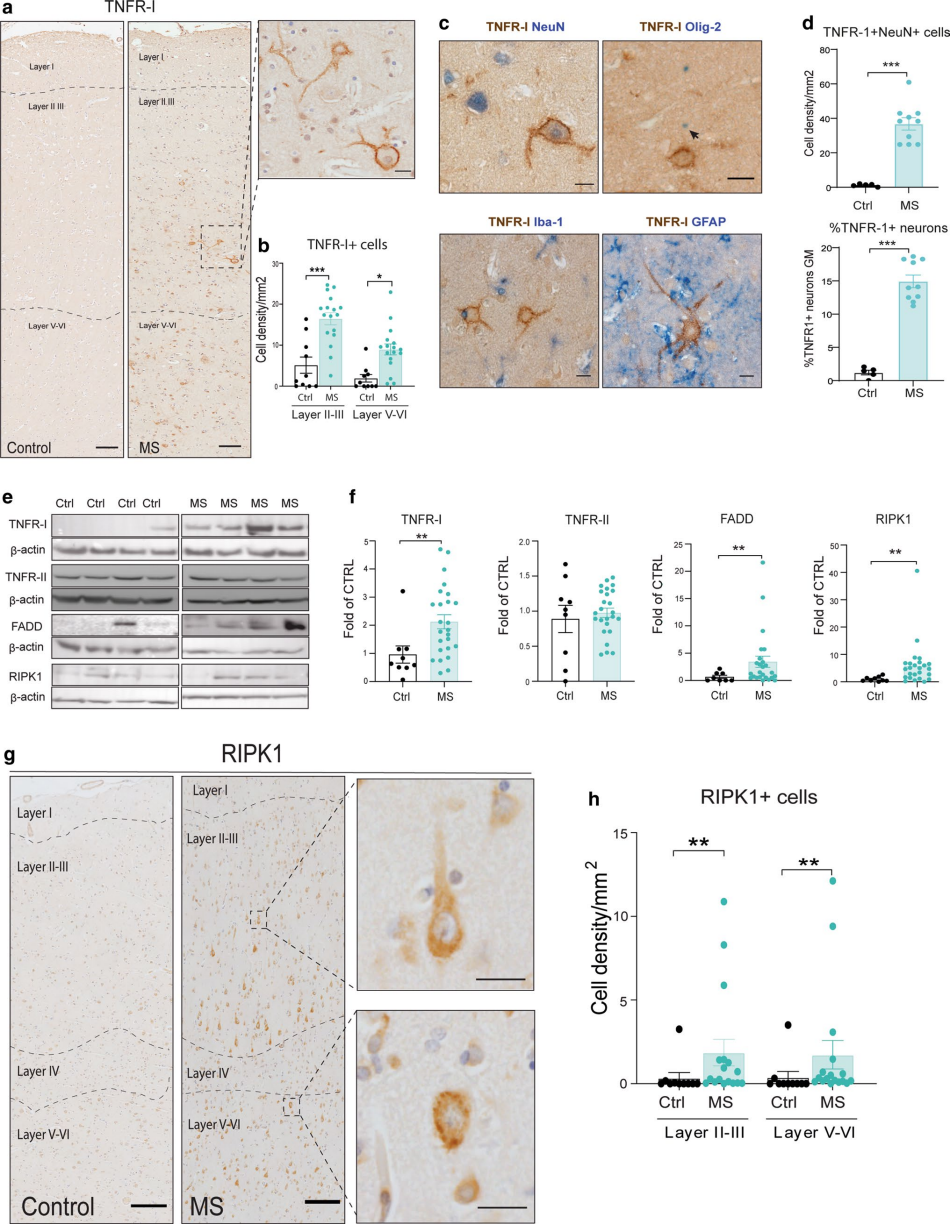
TNFR1 signaling is upregulated in neurons in MS grey matter. **a** TNFR-1 immunohistochemistry in a control and MS case. Insert shows membrane localisation of TNFR1 on macroneurons (scale bars: 200 μm, 20 μm). **b** Quantification of total TNFR1+ cells in MS cases (*n* = 17) and controls (*n* = 10) within the cortical layers II–III and V–VI. **c** Double IHC staining of TNFR-1 with NeuN, GFAP, Iba-1 or Olig-2 in MS cortical GM (scale bar: 20 μm). **d** Quantification of TNFR1+ NeuN+ neuron cell density in the GM of MS cases (*n* = 10) and controls (*n* = 5) and the proportion of neurons expressing TNFR1. **e** Western blot analysis of the protein levels of TNFR-I, TNFR-II, FADD and RIPK1 in the GM of MS cases (*n* = 25) and controls (*n* = 9) and quantification (**f**). Full WB blots are shown in suppl. Fig. 7. **g** RIPK1 IHC staining in a control and MS case. Insets show RIPK1+ macroneurons in layers II–III and V–VI (scale bars: 200 μm, 20 μm). **h** Quantification of the RIPK1+ cell density in MS cases (*n* = 17) and controls (*n* = 10), showing a significant increase in cortical layers II–III and V–VI. All data are represented as mean ± SEM. For two-group comparisons Mann–Whitney test was used and for more than two groups Kruskal–Wallis followed by Dunn’s multiple comparisons test, *p < 0.05, **p < 0.01, ***p < 0.001

A previous transcriptomic analysis of cortical MS GM identified a possible dysregulation in TNF signaling at the mRNA level in secondary progressive MS (SPMS) brains. Given the importance of this finding for understanding the molecular mechanisms of neurodegeneration in MS, we studied the detailed changes in TNF signaling pathways at the protein level in 28 brains from secondary progressive MS patients (SPMS) and ten non-neurological controls (detailed clinical and neuropathological characterization in suppl. Table 1 and suppl. Fig. 1). The cohort of MS brains was chosen to have a wide range of cortical GM demyelination (mean: 30.5 ± 3.3%), perivascular inflammation (mean: 79.9 ± 12.9 cells/mm^2^), meningeal inflammation (mean: 114.0 ± 17.8 cells/mm^2^), microglia activation (mean: 156.0 ± 28.9 HLA-DR+ cells/mm^2^) and neuronal density (mean: 409.5 ± 18.1 HuC/D+ cells/mm^2^) (suppl. Fig. 1a–g). Neuronal loss was evident through all layers but only reached significance in layers II–III (suppl. Fig. 1g). Immunohistochemistry (IHC) demonstrated a substantial and significant upregulation of TNFR1 expression in cortical macroneurons in layers II–III and V–VI in MS GM, with no apparent reactivity in microglia, astrocytes or oligodendrocytes (Fig. 1a–c). Neuronal TNFR1 expression was almost absent in the normal control cortical GM (Fig. 1a). The percentage of total neurons expressing TNFR1 was increased by 12.4-fold in MS and constituted 14.9 ± 3.0% in MS and only 1.2 ± 0.7 in controls (Fig. 1d). TNFR1 protein and mRNA levels were both significantly increased in the GM of MS cases (Fig. 1e, f; suppl. Fig. 2a). In contrast, TNFR-II protein and mRNA levels did not vary between groups (Fig. 1e, f; suppl. Fig. 2a).

Next, we investigated the link between TNFR1 and the activation of cell death by studying the expression of three key initial regulators: Fas ligand associated death receptor (FADD), cylindromatosis (CYLD) and receptor-interacting protein kinase 1 (RIPK1) [43]. A significant upregulation of FADD was found in MS grey matter compared to controls, while CYLD expression was unchanged (Fig. 1e, f; suppl. Fig. 2b). RIPK1 is the key molecular switch for TNF signaling pathways and its protein levels were significantly increased by a mean of sevenfold in MS grey matter, this result being also verified for mRNA levels (Fig. 1e, f; suppl. Fig. 2a). There was a non-significant trend towards the association between RIPK1 protein levels and the extent of cortical demyelination and meningeal inflammation (suppl. Fig. 2c), but no correlation with any clinical outcome variables (not shown). RIPK1 was found to be expressed mainly in a discrete population of neurons spread throughout the entire cortex in MS GM, with only low levels of expression in astrocytes and microglia (Fig. 1g, h; suppl. Fig. 2d). The most striking upregulation was in pyramidal neurons in layers II–III and V–VI of the MS cortex, (Fig. 1h) and it was present as both diffuse cytoplasmic staining and large aggregates (Fig. 1g). In contrast, expression in the control brain was very low (Fig. 1g, h). These results indicate a neuronal-specific activation of TNFR1-mediated signaling in MS GM.

### Cleaved caspase-8 levels and apoptotic signaling are downregulated in MS grey matter

**Fig. 2:**
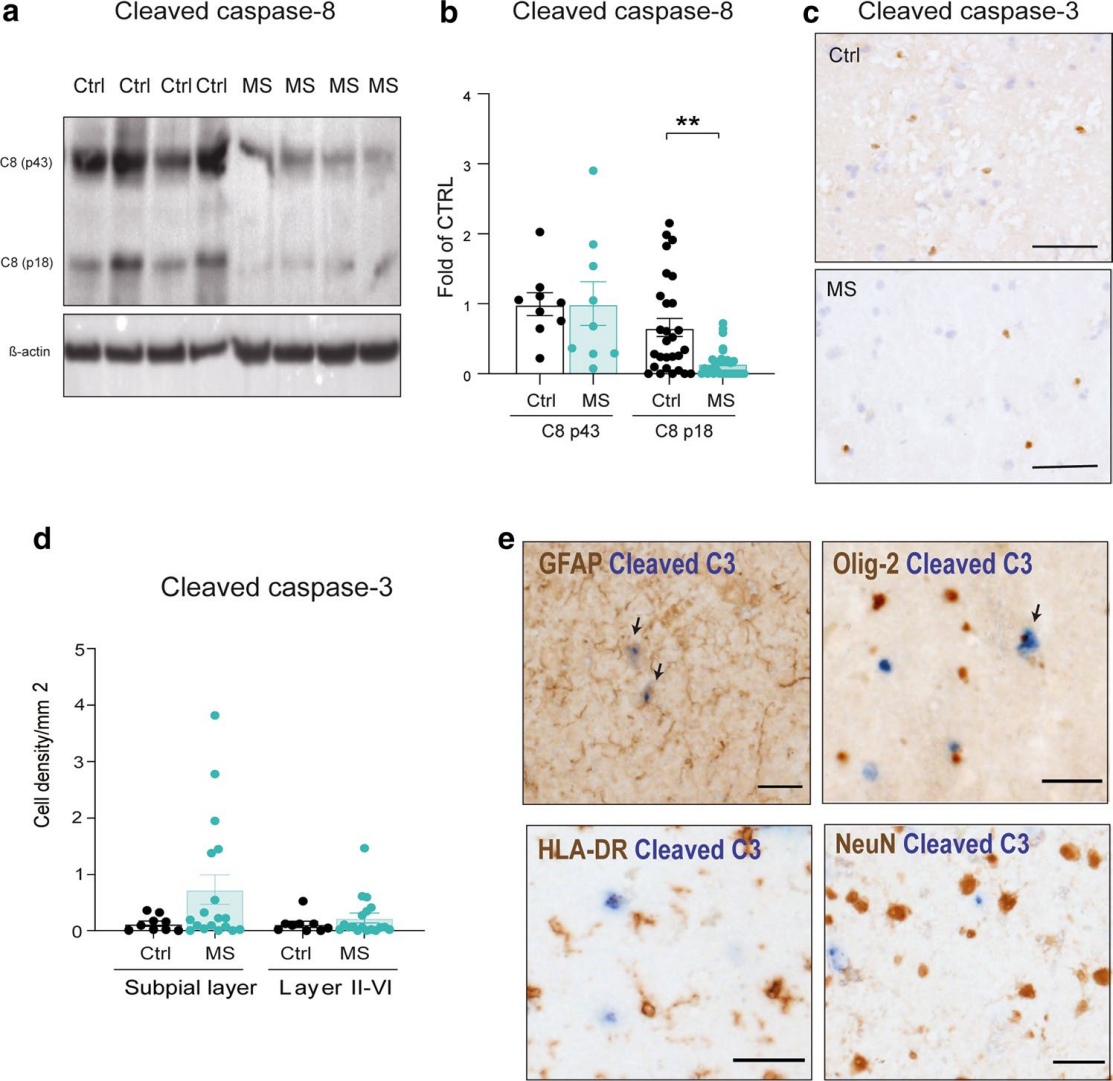
Cleaved caspase-8 levels and apoptosis signaling are downregulated in MS grey matter. **a** Western blot of levels of caspase-8 cleavage, representative images. Full blots are shown in suppl. Fig. 7. **b** Quantitative analysis of the cleaved subunits of caspase-8 (p18 and p43) in tissue lysates from cortical GM in MS (*n* = 23) and control (*n* = 9). **c** Distribution of cleaved caspase-3 immunostaining in MS and control cortical GM (scale bar: 30 mm). **d** Quantification of C-Casp3+ cells in MS cases (*n* = 18) and controls (*n* = 9) in the immediate subpial layer and layers II–IV. **e** Double IHC staining of C-Casp3 (blue) with NeuN, GFAP, HLA-DR or Olig-2 (brown) in MS cortical GM (scale bars: 20 μm). All data are represented as mean ± SEM. Kruskal–Wallis followed by Dunn’s multiple comparisons test, **p < 0.01

To determine whether apoptotic signaling was activated in neurons in MS cortex, we next studied the activation of caspase-dependent apoptosis. The protein levels of the cleaved active p18 subunit were significantly downregulated by 5.3-fold in MS GM, whereas the subunit p43 levels were unchanged (Fig. 2a, b). The p18 subunit activates the downstream pathway of apoptosis, beginning with the cleavage of procaspase-3 into the p17 and p19 subunits. A low level of cleaved caspase-3 (CC3) staining was detected in MS and controls with no significant differences between groups (Fig. 2c, d). Additionally, labelled cells were mainly located in the immediate subpial layer (Fig. 2c, d). In MS GM, the vast majority of CC3+ cells co-expressed the astrocytic marker GFAP, with a small number co-expressing the oligodendrocyte marker Olig-2 (Fig. 2e). NeuN+ CC3+ double-positive neurons were extremely scarce and no differences were found between MS and controls (0.08 ± 0.04% of NeuN+ cells in MS and 0.18 ± 0.14% in controls) (suppl. Fig. 3a, b). These results indicate the downregulation of caspase-8-dependent apoptosis in neurons in MS.

### Activation of necroptosis in MS cortical neurons

**Fig. 3:**
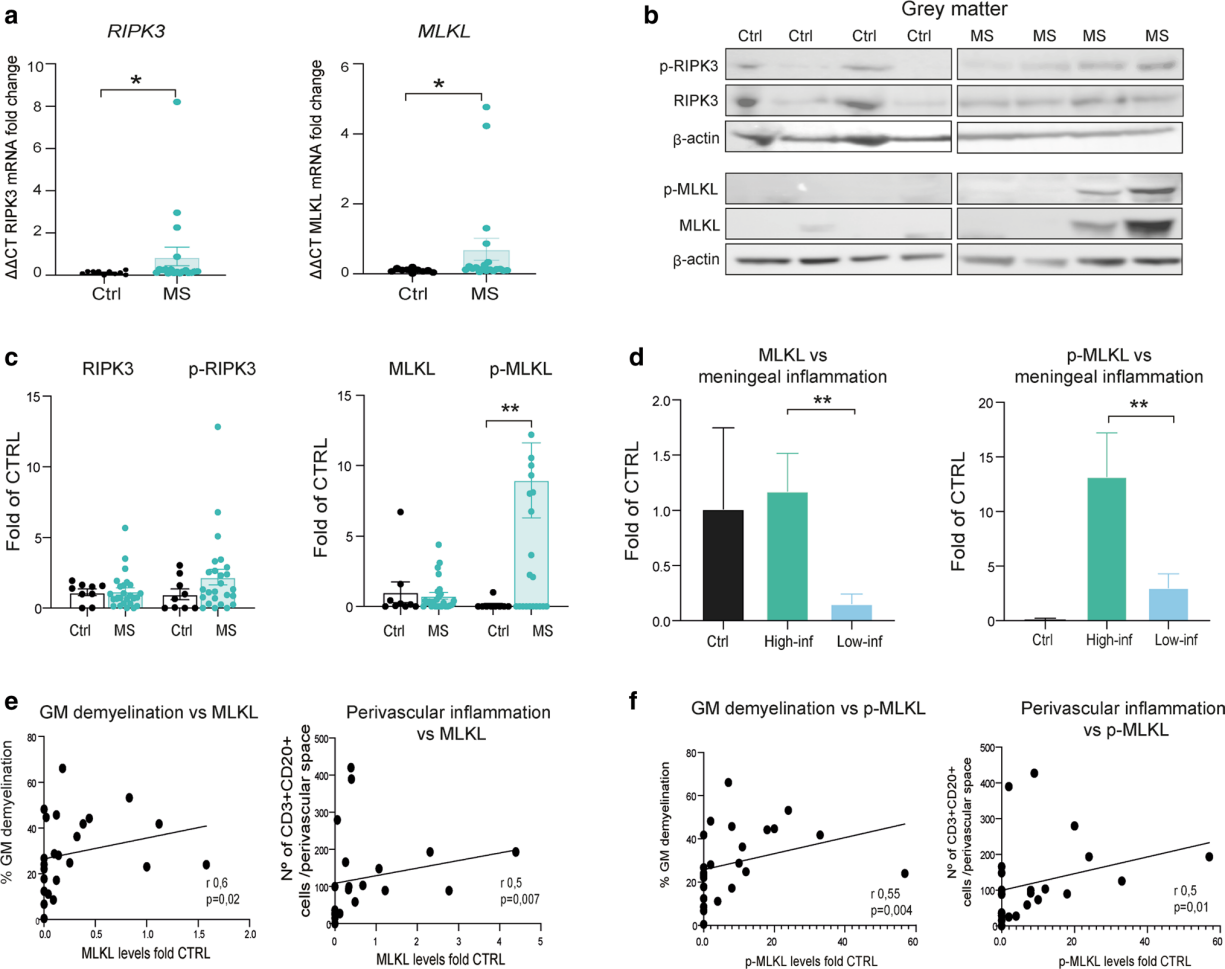
Activation of necroptosis signaling in MS cortical grey matter. **a** Analysis of mRNA levels for the RIPK3 and MLKL genes in cortical GM in MS cases (*n* = 19) and controls (*n* = 10). **b** Representative Western blots of RIPK3/p-RIPK3 and MLKL/p-MLKL. Full blots are shown in suppl. Fig. 7. **c** Quantification of RIPK3 and pRIPK3 (left) and MLKL and pMLKL (right) protein levels in MS (*n* = 25) and control cases (*n* = 9), normalized to β-actin. **d** Analysis of MLKL and pMLKL protein levels in high and low meningeal inflammation MS cases, as defined in the methods (*n* = 15 high, *n* = 10 low), and controls (*n* = 9). **e** Correlation analysis of MLKL protein levels and the percentage of grey matter demyelination and degree of perivascular inflammation (CD3+ CD20 cell numbers) (*n* = 24). **f** Correlation analysis of pMLKL protein levels and the percentage of grey matter demyelination and degree of perivascular inflammation (CD3+ CD20 cell numbers) (*n* = 24). For two group comparisons Mann–Whitney test was used and for more than two groups Kruskal–Wallis followed by Dunn’s multiple comparisons test. Correlation analysis by Spearman comparison. Data are represented as mean ± SEM, *p < 0.05, **p < 0.01

We next asked whether necroptotic signaling was activated in cortical neurons in MS, which relies on the phosphorylation of first RIPK3 and then MLKL. We found an increase in the mean gene expression for both RIPK3 and MLKL in MS cortex, 2.2- and 2.3-fold, respectively (Fig. 3a). The levels of the non-activated RIPK3 and MLKL proteins were not significantly altered (Fig. 3b, c). However, pMLKL protein levels were remarkably 89.3-fold upregulated in the grey matter of MS cases compared to control (MS: 8.93 ± 2.65; Ctrl: 0.1 ± 0.1), together with a trend towards increased levels of pRIPK3 (MS: 2.2 ± 0.55; Ctrl: 0.9 ± 0.4) (Fig. 3b, c). PhosphoMLKL expression in MS cortex appeared to be highly heterogenous, suggesting that pMLKL levels could be related to the microenvironment in individual brains (Fig. 3c). When MS cases were classified according to the severity of meningeal inflammation, pMLKL and MLKL levels were both significantly increased in cases with more abundant meningeal inflammation when compared to those with low levels of meningeal inflammation (6.5- and 8.0-fold, respectively) (Fig. 3d). Significant moderate correlations were observed between the levels of both MLKL and pMLKL and cortical demyelination and inflammation (Fig. 3e, f).

**Fig. 4:**
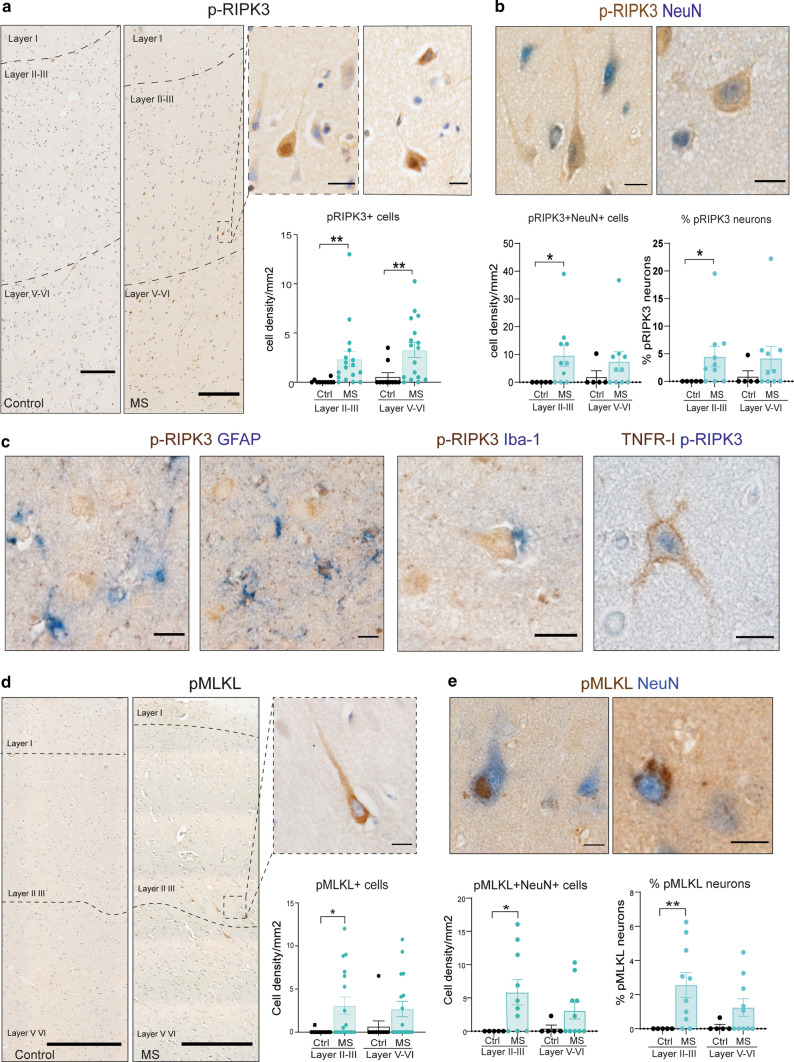
p-RIPK3 and p-MLKL are upregulated in cortical neurons in MS cases. **a** Representative images of pRIPK3 IHC in the cortical GM from a control and MS case (scale bar = 200 μm) and the quantification of pRIPK3+ cells in MS (*n* = 17) and control (*n* = 10) cases within layers II–III and V–VI (scale bars: 200 μm, 20 μm). **b** Double IHC staining of pRIPK3 (brown) with NeuN (blue) (scale bar: 20 μm) and the quantification of the density of pRIPK3+ NeuN+ cells and the proportion of neurons expressing pRIPK3 within layers II–III and V–VI in MS cases (*n* = 10) and controls (*n* = 5). **c** Representative images of double IHC staining of pRIPK3 with GFAP, Iba-1 and TNFRI in MS cortical GM (scale bar: 20 μm). **d** Phospho-MLKL IHC in a control and MS case, with insert showing the pyramidal morphology of a pMLKL+ neuron (scale bar: 200 μm, 20 μm), and quantification of the cell density of pMLKL+ cells in layers II/III and V/VI of the cortical grey matter in MS (*n* = 17) and control (*n* = 10) cases. **e** Double IHC staining of pMLKL with NeuN, showing clear cytoplasmic and nuclear/perinuclear localisation (scale bar: 20 μm). Quantification of the density of pMLKL+ NeuN+ cells (left) and the proportion of NeuN+ neurons expressing pMLKL (right) within layers II–III and V–VI in MS cases (*n* = 10) and controls (*n* = 5). Kruskal–Wallis followed by Dunn’s multiple comparisons test. Data are represented as mean ± SEM, *p < 0.05, **p < 0.01

Within the cortical layers, pRIPK3-expressing cells morphologically resembled pyramidal neurons and their density was increased in both layers II–III and V–VI in MS compared to controls (Fig. 4a). Such cells were rarely seen in the control brain. Immunolabeling was mainly diffuse in the cytoplasm together with some nuclear localisation (Fig. 4a). The cellular identity of the majority of pRIPK3-positive cells was confirmed as neuronal using double immunohistochemistry with NeuN (4.36% ± 1.98% of total NeuN+ neurons in MS; 0.56 ± 0.47% in control brain) (Fig. 4b). Some low-level immunoreactivity was also seen in astrocytes, but none in microglia (Fig. 4c). Co-localisation of pRIPK3 together with TNFR1 (Fig. 4c) strongly suggests a TNFR1-mediated activation of necroptosis signaling in cortical neurons in MS GM. Immunohistochemistry to further validate the expression of pMLKL in the GM of MS cases demonstrated an 8.6-fold increase in the number of pMLKL+ cells in comparison to controls (Fig. 4d). Indeed, a small number of pMLKL+ neurons were only seen in one of the control brains (Fig. 4d). The highest numbers of pMLKL+ neurons were found in layers II–III, with a 30-fold increase (Fig. 4d). In line with our findings for RIPK1 and pRIPK3, double immunohistochemistry revealed that pMLKL was mainly expressed by NeuN+ pyramidal cortical neurons in the GM, with 1.98% ± 0.5% of total neurons expressing pMLKL in MS cases compared to 0.06% ± 0.06% in controls (Fig. 4e). We next investigated whether the surrounding inflammatory microenvironment was associated with the presence of pMLKL in neurons. We found a strong significant correlation with the number of HLA-DR+ (a marker of activated microglia/macrophages) cells in the GM (*r* = 0.6; *p* = 0.01) and a weaker non-significant correlation with HLA-DR+ cells within the meninges (*r* = 0.4; *p* = 0.09) (suppl. Fig. 4).

### Necrosome formation occurs in the MS cortex

**Fig. 5:**
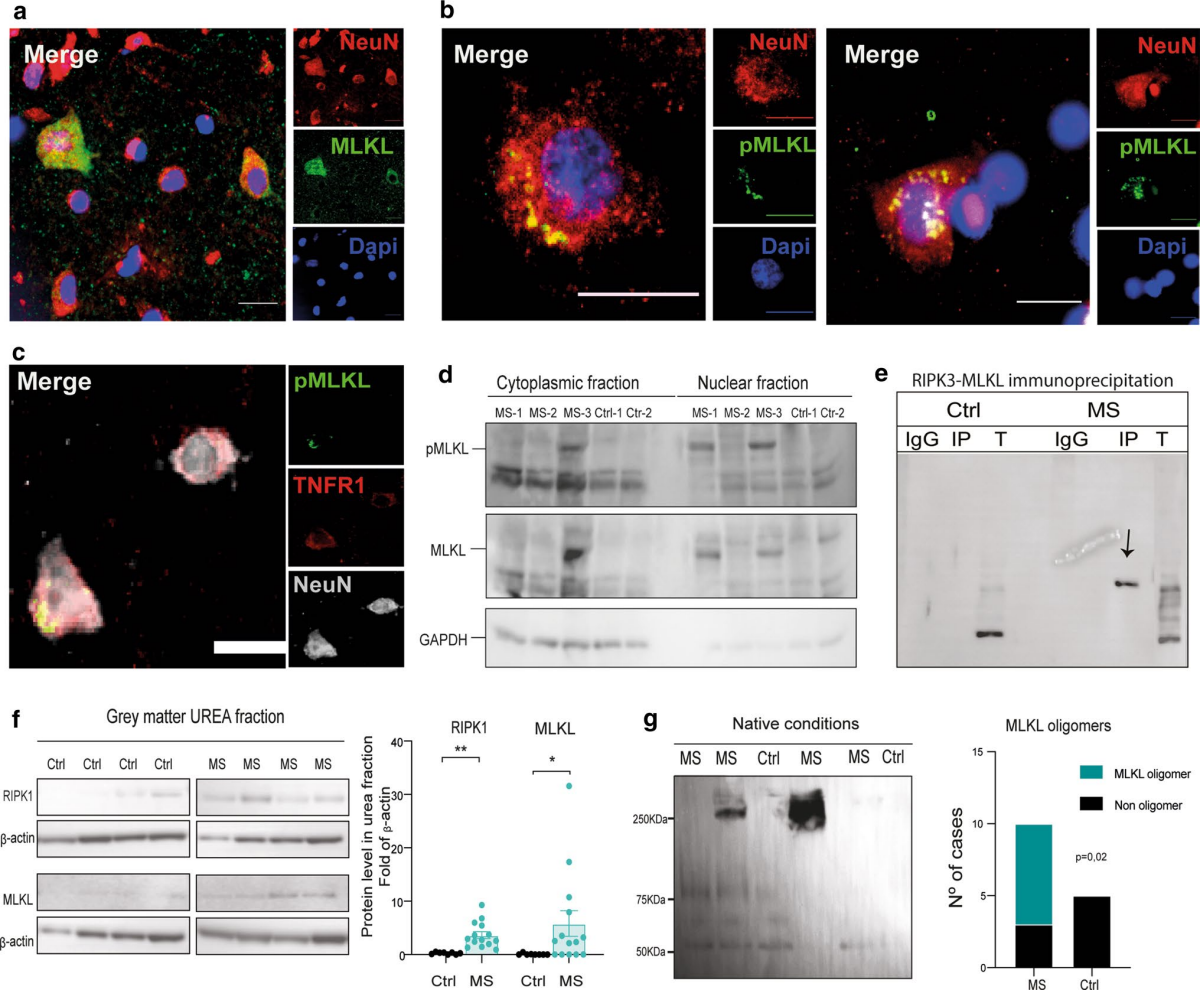
MLKL activation and necrosome formation in MS cortical grey matter. **a** Immunofluorescence imaging of MLKL (green) in NeuN+ neurons (red) with DAPI+ cell nuclei (blue) in the grey matter of an MS case (scale bar: 20 μm). **b** Examples of immunofluorescence for p-MLKL (green) in NeuN+ neurons (red; Dapi, blue) in MS GM, showing necrosome-like accumulations in the cytoplasm (scale bar: 20 μm). **c** Immunofluorescence for p-MLKL (green), TNFR1 (red) and NeuN+ (grey) in neurons in MS GM (scale bar: 20 μm). **d** WB analysis of MLKL and p-MLKL levels in the cytoplasmic and nuclear fraction of GM tissue from three MS cases and two controls. **e** Immunoprecipitation analysis of RIPK3 and co-IP with MLKL in the GM of a control and MS case. **f** WB analysis of RIPK1 and MLKL from the urea soluble fraction of tissue lysates of MS GM (*n* = 14) and controls (*n* = 7), normalized to β-actin. Full blots are shown in suppl. Fig. 7. **g** WB analysis of MLKL from GM tissue lysate extracted in native conditions, showing the presence of oligomers. Quantification of the presence of MLKL tetramers (250 KDa) in MS GM (*n* = 10) compared to controls (*n* = 5). For two group comparisons the Mann–Whitney test was used and for more than two groups Kruskal–Wallis followed by Dunn’s multiple comparisons test. Data are represented as mean ± SEM, *p < 0.05, **p < 0.01

Upon phosphorylation, MLKL is reported to oligomerize in the cytoplasm and migrate to the plasma membrane, where it causes membrane rupture and ultimately cell death. Both MLKL and pMLKL were variably expressed in both the cytoplasmic and nuclear/perinuclear compartment in neurons (Fig. 5a, b), which was validated by subcellular fractionation and WB (Fig. 5d). Importantly, we found pMLKL protein co-localizing with TNFR1 in neurons, supporting a link between TNFR1 and necroptosis activation in MS (Fig. 5c). In addition, previous studies have shown that the necrosome complex, consisting of RIPK1, RIPK3 and MLKL, assembles as a fibrillar structure that can be extracted by chaotropic agents such as urea [28]. In keeping with this, we found that RIPK3 co-immunoprecipitated with MLKL in MS GM but not in controls (Fig. 5e) and we identified the presence of RIPK1 and MLKL in the urea soluble fraction by WB and this was markedly increased in MS samples (Fig. 5f). Using non-reducing conditions to probe the oligomeric state of MLKL, we found the presence of MLKL oligomers of approximately 250 KDa only in the MS cortex, most likely representing tetramers (Fig. 5g).

### The number of pMLKL expressing neurons is associated with more rapid disease progression

**Fig. 6:**
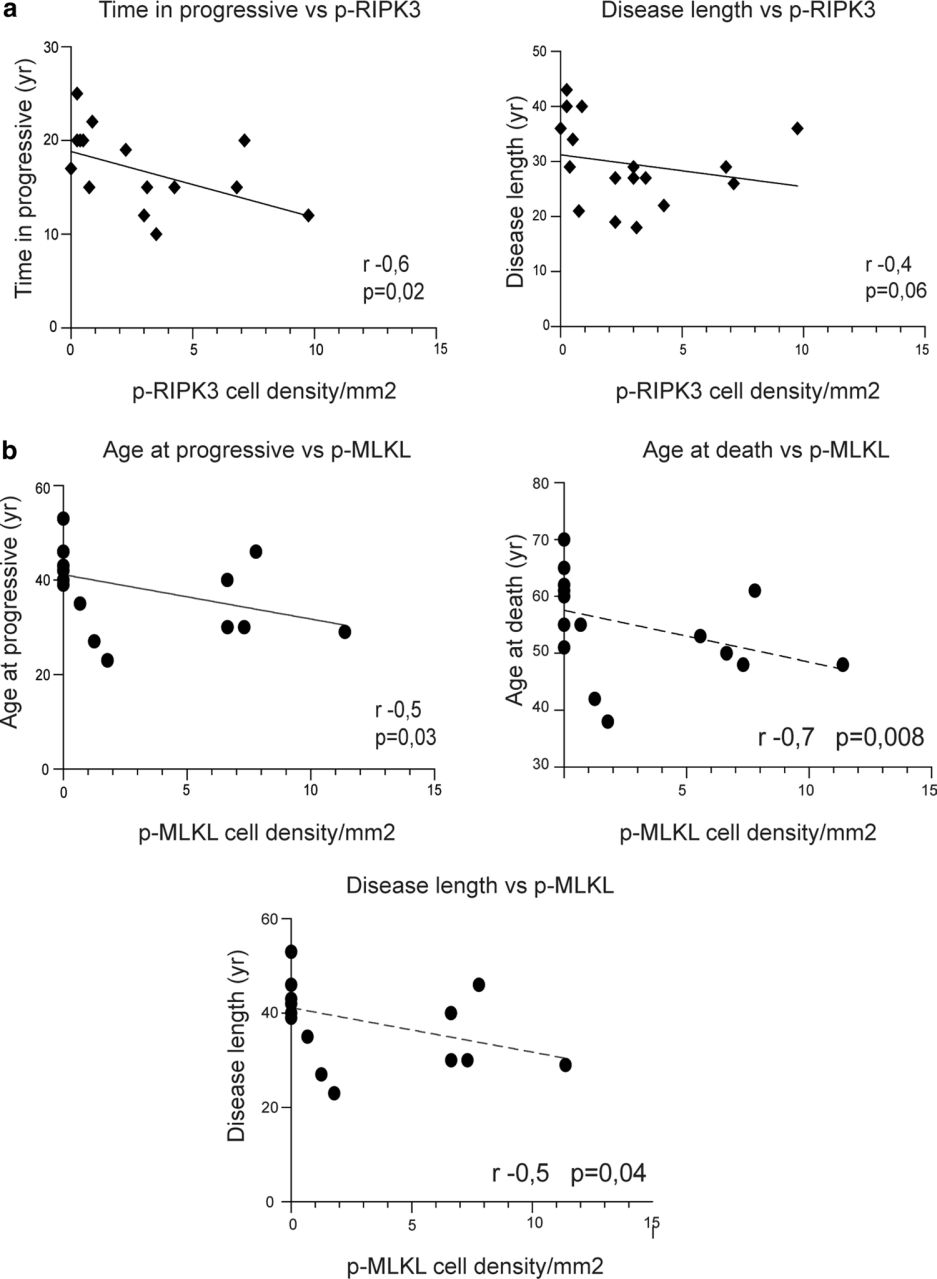
The number of pMLKL and pRIPK3 cells associated with a more rapid disease progression. **a** Correlation analysis between the number of pRIPK3+ cells in cortical GM and the time in progression and clinical disease length in MS cases (*n* = 17). **b** Correlation analysis between the number of pMLKL+ cells in cortical GM and the age at progression, age at death and clinical disease length in MS cases (*n* = 17). Correlation analysis by Spearman comparison

To determine the relationship between the activation of necroptosis in neurons and disease progression, we correlated the density of neurons expressing pRIPK3 and pMLKL with clinical parameters (Fig. 6). The density of pRIPK3+ cells correlated inversely with the time in the progressive phase of the disease (*r* = − 0.6; *p* = 0.02) and there was a similar non-significant trend with the disease length (*r* = − 0.4; *p* = 0.06) (Fig. 6a). We further compared the clinical variables with the density of pMLKL+ neurons and found strong significant inverse correlations between the density of pMLKL+ cells and the age at progression (*r* = − 0.55; *p* = 0.03), the age at death (*r* = − 0.7; *p* = 0.008) and the disease length (*r* = − 0.5; *p* = 0.04) (Fig. 6b).

### Chronic exposure to TNF and IFNγ in the CSF induces neurodegeneration

To determine whether TNF-mediated induction of necroptotic neurodegeneration could be induced by chronic expression of pro-inflammatory cytokines in the meningeal compartment, we used a novel animal model in which lentiviral vectors containing the transgenes for human TNF and IFNγ were injected into the brain midline subarachnoid space of Dark Agouti (DA) rats, thereby inducing meningeal cells to persistently produce these cytokines. Previous studies had ascertained that it was necessary to combine TNF with IFNγ to induce TNFR1 expression within CNS cells [13, 21]. After 28 days post injection (dpi), we observed the presence of leukocyte aggregation in the sagittal sulcus in rats injected with cytokine vectors (suppl. Fig. 5a, b), with meningeal infiltrates enriched in CD4/CD8 T-lymphocytes and CD79a B-lymphocytes. Injected rats displayed an increased number of microglial cells (Iba-1+ cells) in the GM upper layers with no evident changes in the number of astrocytes (GFAP+ cells) (suppl. Fig. 5c, d). These results were accompanied by a significant 20% decrease in the number of neurons located in cortical layer II, with smaller non-significant decreases in the deeper cortical layers III and V (suppl. Fig. 5e).

**Fig. 7:**
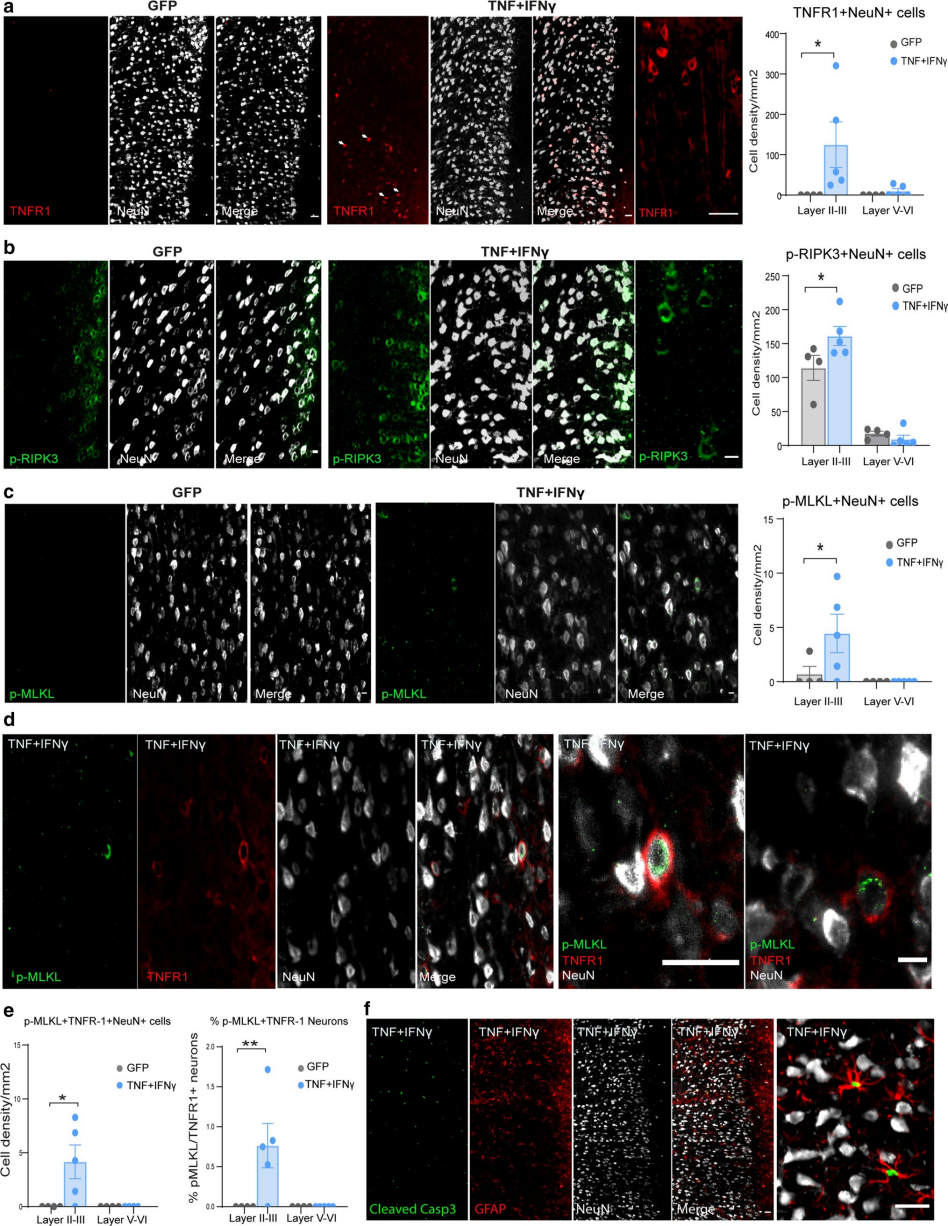
Chronic exposure to TNF and IFNγ induces activation of necroptosis in neurons. **a** Representative immunofluorescence images for expression of TNFR1 in NeuN+ neurons in GFP vector (*n* = 4) and TNF+ INFγ vector injected animals at 28 dpi (*n* = 5). The cell density of TNFR-I+ NeuN+ positive cells was quantified in layers II–III and V–VI in both animal groups (scale bar: 200 μm, 20 μm). **b** Representative immunofluorescence images for expression of pRIPK3 in NeuN+ neurons in GFP vector animals (*n* = 4) and TNF+ INFγ vector injected animals at 28 dpi (*n* = 5) and quantification of pRIPK3+ NeuN+ cell density in layers II–III and V–VI (scale bar: 200 μm, 20 μm). **c** Representative immunofluorescence images for pMLKL expression in NeuN+ neurons in GFP vector animals (*n* = 4) and TNF+ INFγ vector injected animals at 28 dpi (*n* = 5) and quantification of pMLKL+ NeuN+ cell density in layers II–III and V–VI (scale bar: 200 μm, 20 μm). **d** Representative immunofluorescence images for pMLKL and TNFR1 expression in NeuN+ neurons in GFP vector animals (*n* = 4) and TNF+ INFγ vector injected animals 28 dpi (*n* = 5). **e** Quantification of pMLKL+ TNFR1+ NeuN+ cell density and the percentage of neurons expressing both markers in layers II–III and V–VI in GFP vector animals (*n* = 4) and TNF+ INFγ vector injected animals at 28 dpi (*n* = 5) (scale bar: 200 μm, 20 μm). **f** Representative images of sections from TNF+ INFγ vector injected animals immunostained for cleaved caspase-3 (green), GFAP (red) and NeuN (grey) (scale bar: 20 μm). One-way ANOVA with Bonferroni’s post-hoc correction. Data are represented as mean ± SEM, *p < 0.05, **p < 0.01, ***p < 0.001

In TNF/IFNγ vector injected animals, TNFR1+ NeuN+ neurons were observed predominantly in the upper cortical subpial layers (Fig. 7a). Quantitative analysis of the TNFR1+ NeuN+ neurons after 28 dpi showed a significant 125-fold increase within layers II/III (Fig. 7a). Negligible numbers of TNFR1+ cells were seen in the cortical layers of the GFP vector control group. An increase in pRIPK3+ NeuN+ and pMLKL+ NeuN+ neurons was also seen in layers II–III (1.7- and 7.9-fold, respectively), whereas pMLKL+ cells were rarely present in GFP vector injected animals (Fig. 7b, c). Triple immunofluorescence staining for pMLKL, TNFR1 and NeuN demonstrated the co-localization of the three markers only in TNF/IFNγ vector injected animals, with a significant increase and higher proportion of neurons co-localizing pMLKL and TNFR1 in layers II–III, but not layers V–VI (Fig. 7d, e). Immunofluorescence images for MLKL and pMLKL with the neuronal marker NeuN showed that neurons were expressing MLKL within both the cytoplasm and nucleus, whereas pMLKL staining was in large aggregates within the cytoplasm (suppl. Fig. 5f, g). In keeping with the human tissue results, cleaved caspase-3 immunoreactivity was only observed in GFAP+ astrocytes and not in NeuN+ neurons (Fig. 7f). Thus, the persistent production of TNF and INFγ in the cerebral subarachnoid space of DA rats induces neurodegeneration associated with an increase in expression of necroptotic signaling molecules in neurons in the underlying upper cortical layers.

### In vitro stimulation with TNF induces necroptosis activation in cortical neurons when apoptosis is inhibited

**Fig. 8:**
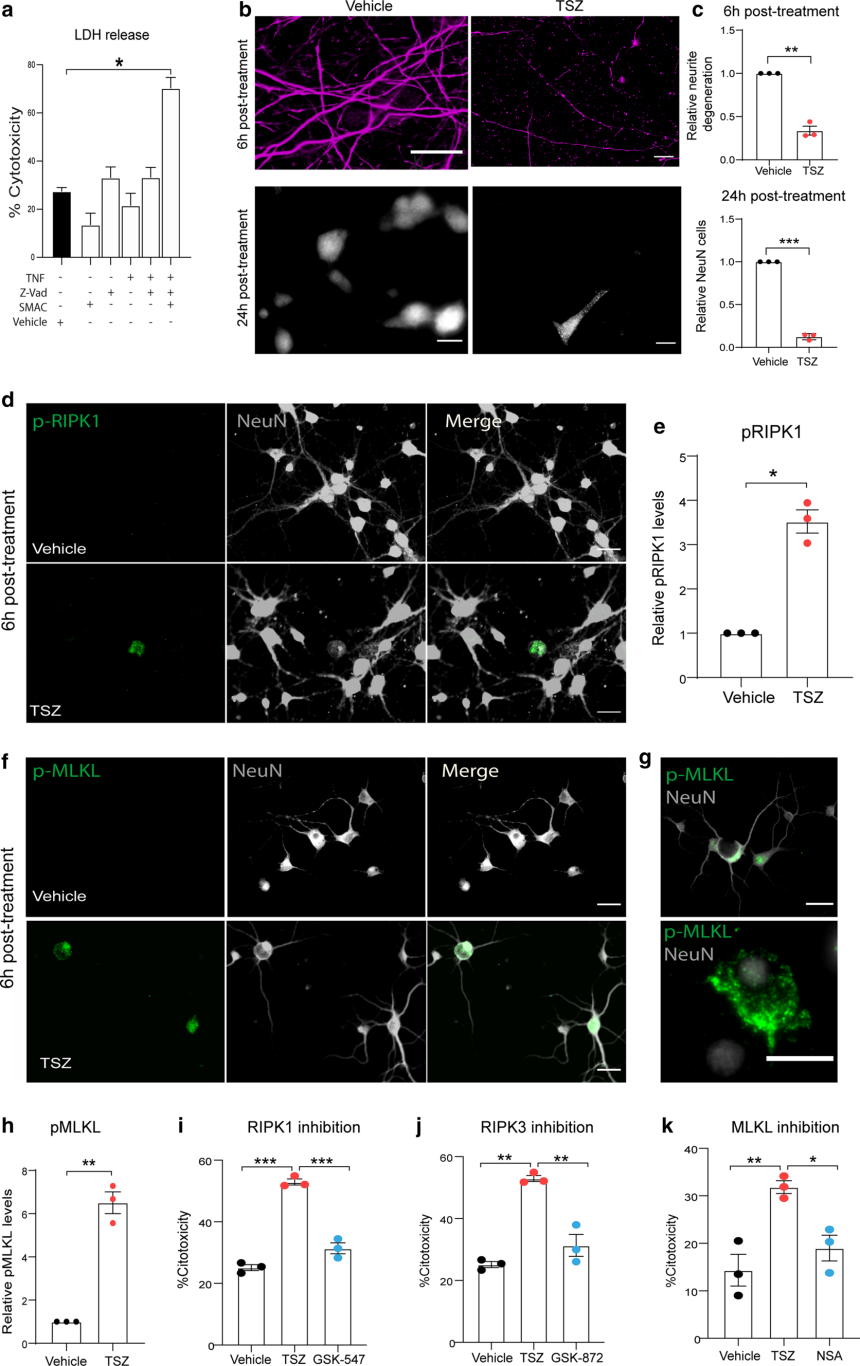
TNF mediated activation of necroptosis in primary cortical neurons when apoptosis is inhibited. **a** Primary cortical neurons were treated with combinations of rat TNF (100 ng/ml), SMAC mimetic (2 μM) Z-vad (10 μM) and vehicle control and viability assessed by measuring LDH release at 24 h. **b** Representative images of cultured primary cortical neurons treated with TSZ or vehicle (DMSO) immunostained for NFH-200 (upper panels, 6 h post treatment) and NeuN (lower panels, 24 h post treatment) (scale bar: 20 μm). **c** Quantification of NFH-200 fluorescence intensity as a measure of neurite degeneration (6 h post treatment) and NeuN+ neurons (24 h post treatment) (3 replicates per group). **d** Representative immunofluorescence images for pRIPK1 expression in NeuN+ cortical neurons treated with TSZ and vehicle (scale bar: 20 μm) and their quantification (**e**) (3 replicates per group). **f** Representative immunofluorescence images for pMLKL expression in NeuN+ cortical neurons treated with TSZ and vehicle for 6 h. (scale bar: 20 μm). **g** Microphotographs showing accumulation of pMLKL protein close to the plasma membrane in cortical neurons treated with TSZ after 6 h. **h** Quantification of pMLKL fluorescence intensity in TSZ and vehicle groups (3 replicates per group). Primary cortical neurons treated with and without GSK-547 (10 nM) (**i**), GSK-872 (100 nM) (**j**) and necrosulfonamide (NSA; 1 μM) (**k**) and viability assessed by measuring LDH release (*n* = 3 replicates per group). One-way ANOVA with Bonferroni’s post-hoc correction. Data are represented as mean ± SEM, *p < 0.05, **p < 0.01, ***p < 0.001

To evaluate the activation of necroptosis by TNF in cortical neurons, we exposed dissociated primary cortical neurons to TNF (100 ng/ml) and analysed TNF-mediated cytotoxicity at 24 h by measuring the release of lactate dehydrogenase (LDH) and neurodegeneration by the expression of NeuN and the 200 KDa heavy chain of neurofilament protein (NfH). We did not find any effect of TNF alone on the parameters measured (Fig. 8a; suppl. Fig. 6a, b), suggesting that acute TNF release alone is not sufficient to trigger degeneration of cortical neurons or their neurites. In line with previous reports for non-CNS cells, only when TNF was combined with the caspase inhibitor z-VAD (10 μm) and a SMAC mimetic (SMAC; 2 μm) that degrades the inhibitor of apoptosis proteins (cIAPs), which are a family of antiapoptotic proteins that regulate NF-κB via their E3 ubiquitin ligase activity, did TNF cause a time dependent increase in LDH release, that reached its maximum peak after 24 h of incubation (TSZ treatment) (Fig. 8a; suppl. Fig. 6c). Furthermore, we analysed neurite degeneration after 6 and 24 h of TSZ treatment and we found substantial neurite fragmentation and beading at 6 h post treatment and at 24 h a complete loss of neurites that was accompanied by a decrease in the number of intact neuronal cell bodies (Fig. 8b, c; suppl. Fig. 6d). To determine the activation of necroptosis, we analysed the expression of pRIPK1 (Fig. 8d) and pMLKL (Fig. 8f, g) by immunofluorescence after 6 h of treatment and found an upregulation of these key proteins in cortical neurons in response to TSZ (Fig. 8e, h). PhosphoMLKL was detected in the cell cytoplasm and also close to the plasma membrane and in some neurites (Fig. 8g; suppl. Fig. 6f). In addition, we also found an increase in the protein levels of MLKL in the treated group (suppl. Fig. 6e). To further confirm the role of necroptosis in inducing neuronal cell death after TSZ exposure, we pharmacologically inhibited RIPK1 (GSK-547, 10 nM), RIPK3 (GSK-872; 100 nM) and MLKL (Necrosulfonamide; NSA 1 μM). Inhibition with all three compounds significantly prevented cell cytotoxicity after 24 h of treatment and brought the cytotoxicity levels back to control values (Fig. 8i–k). Taken together these results demonstrate that TNF can trigger necroptotic cell death in cortical neurons through MLKL activation, but only in conditions in which the alternative TNF-signaling pathways have been inhibited.

## Discussion

Neurodegeneration in the MS cortex has been closely linked to a compartmentalised immune response [3, 6] that results in an increasingly inflammatory milieu in the CSF bathing the cortical layers [34]. This leads directly and indirectly to pathological changes in the underlying cortical GM [14, 21, 33, 35]. We have previously reported that elevated levels of soluble TNF in MS CSF associate with increased cortical lesions, CSF neurofilament levels and atrophy [34, 38]. The present study links a local increase in cortical GM neuronal TNFR1 expression to neurodegeneration via necroptosis signaling in progressive MS, in the presence of reduced apoptotic signaling. Chronic in vivo augmentation of TNF and INFγ in the meningeal space of the rat recapitulates these features of neuronal necroptosis, ultimately inducing neurodegeneration. Lastly, we show that primary cortical neurons in vitro undergo TNF induced and RIPK1/RIPK3/MLKL dependent necroptosis, but only when caspase 8 induced apoptosis and NFκB signaling are both inhibited.

TNF signaling pathways have a complex and multifaceted role in the pathogenesis of MS, mainly due to differential receptor binding of the soluble and membrane-bound forms and activation of pleiotropic signaling pathways. In the brain parenchyma, tmTNF interaction with TNFR2 promotes oligodendrocyte regeneration, CNS remyelination and neuroprotection [1, 47, 57], whereas binding of sTNF to TNFR1 is generally involved in the initiation of cytotoxic and pro-inflammatory processes [46, 54]. Our data showing that TNFR1 is upregulated by a proportion of cortical neurons, in association with the TNFR1-associated adaptor proteins, FADD and RIPK1, indicates that TNF may be responsible for initiating cell death mechanisms in MS. A decrease in caspase-8 cleavage, together with the increase in MLKL and RIPK3 protein phosphorylation and necrosome formation, indicates that the balance in TNF signaling is switched towards the execution of necroptosis, away from apoptosis [33, 42]. Caspase 8 levels make a major contribution to the susceptibility of cells to necroptosis and this is also likely to be the case for neurons. The downregulation of cleaved caspase 8 p18 levels has previously been demonstrated in MS WM [43] and now in MS GM in our study. This in turn leads to a downregulation of caspase-dependent apoptosis, as demonstrated by the rarity of activated caspase-3 expressing neurons in this study. Activated caspase 8 cleaves the kinase domain of RIPK1 and directs TNFR1 signaling towards apoptosis, and so when caspase 8 levels are reduced, RIPK1 kinase activity remains intact and directs signalling towards necroptosis via autophosphorylation and then phosphorylation of RIPK3 and MLKL [15, 24, 55, 59]. Increased levels of cFLIP_L_ can block activation of caspase 8 [42], but would then promote ubiquitination of RIPK1 and NFkB signaling and cell survival, rather than necroptosis, which does not appear to be the case in MS GM [33]. The factors contributing to decreased caspase 8 activation in chronic inflammatory conditions have yet to be described. However, it is also important to note that a decrease in casapse 8 levels is not a pre-requisite for activation of necroptosis, as over-expression of RIPK3 can overcome this requirement [64].

When dissociated rat cortical neurons were treated with TNF, they only became susceptible to the induction of necroptosis in the presence of antagonists of the inhibitor of apoptosis proteins (XIAP, cIAP1, cIAP2) and inhibition of caspases, which directs TNF action towards necrosome formation. This is also in keeping with our data from both human tissues and the rat model, which shows that caspase-dependent apoptotic signaling was downregulated. Thus, in the MS cortex the chronic exposure to inflammatory stimuli, metabolic stress, ROS and glutamate excitotoxicity might act synergistically to make neurons more vulnerable to TNF [62, 66]. However, our finding that small molecule inhibitors of RIPK1, RIPK3 and MLKL could provide complete protection against TNF stimulated necroptotic neuron death in vitro, suggests that pharmacological manipulation of neuronal necroptosis may be a valid in vivo and clinical approach to reduce this vulnerability of cortical neurons.

The localization of neurons expressing TNFR1, RIPK1, pRIPK3 and pMLKL was very similar and occurred mainly in layers II–III and V–VI, the main pyramidal cell layers of the human cortex. The almost total lack of expression of TNFR1 associated necroptosis signaling molecules seen in the control brains clearly demonstrates that this signaling is induced in response to a pathological change in the microenvironment. The increase in pMLKL protein levels and cell numbers in layers II–III is in keeping with the higher degree of neuronal loss observed in this area in our cohort and in other studies [35, 52]. Neurons expressing RIPK1, pRIPK3 and pMLKL morphologically resembled large pyramidal neurons, which suggests that specific neuronal subtypes found within layer II–III or V–VI could be differentially vulnerable to the inflammatory microenvironment, in keeping with a recent single nuclear RNASeq study of MS cortical GM [52]. The frequency of neurons expressing the activated necroptotic proteins, pRIPK3 and pMLKL, at this single snap-shot in time was low (a mean of < 5% of total neurons), but this would be in keeping with a slow degenerative process that may extend over decades and fits with the slow increase in GM atrophy on MRI [4, 10], together with other slowly evolving processes in the MS brain [20]. Even if we take into account the fact that we do not know how long the neurons express pRIPK3 and pMLKL before they eventually die, whether the rate of neuronal death is constant during the disease duration and whether all neurons expressing pMLKL eventually die, it is still likely that the percentage of necroptotic neurons that we observed could indeed account for the totality of neuronal loss over the disease course. Our results show that neuronal apoptosis if it occurs at all, is very rare in the MS cortex. Although we cannot exclude that other forms of cell death, such as ferroptosis or pyroptosis, may be playing a role, it is unlikely to be a major one.

One of the characteristics of expression of TNF and necroptosis signalling molecules in our study was the high degree of variability in values for the levels of single proteins, at both the protein and gene levels. This is not unexpected given the extreme heterogeneity seen in MS pathology, from mild to rapidly progressing cases, and fits with previous studies showing the correlation between neuronal loss and clinical severity [35, 49]. Here we included cases with both low levels of inflammatory, demyelinating and neurodegenerative pathology and those with high levels so that it was not biased towards more extreme cases. Despite this variability, we found significant differences in TNFR1, FADD and RIPK1 levels between MS and control samples. No significant correlations were found when comparing TNFR1 and FADD levels with the clinical and neuropathological parameters of the MS patients. Such correlations are unlikely between a single molecular entity and gross clinical outcome measures unless an individual molecule is playing a large and direct role in the pathogenesis, as suggested by the positive correlations between the levels of the final executors of cell death, pRIPK3 and pMLKL, and clinical outcomes.

Previous reports have also found evidence of necroptosis activation in MS brains, however, these studies were focused on white matter lesions and did not look at neurons [30, 42]. Necroptosis was also shown to be involved in WM oligodendrocyte degeneration and its inhibition prevented oligodendrocyte death in two models of MS [42]. Microglia in the WM have also been suggested to undergo necroptosis, and this may be a critical step in myelin regeneration following demyelination [30]. However, while we could find signs of some low-level expression of RIPK1 and pRIPK3 in microglia and astrocytes, only neurons were clearly expressing pMLKL in the GM. The presence of RIPK1 and pRIPK3 in glial cells could indicate that these proteins are also involved in triggering neuroinflammation, independent of a role in executing necroptosis [62, 64]. In addition to the direct stimulation of neuron cell death, necroptosis is suggested to also be involved in axon degeneration [44]. In support of this hypothesis, TSZ treatment of rat cortical neurons in this study resulted in neurite degeneration and beading at early time points, together with the expression of pMLKL by neurites. However, the finding of large aggregates of pMLKL in the cell soma at the same time suggests that both processes may be occurring concomitantly.

Our data suggest that CNS cells are susceptible to necroptosis under inflammatory conditions, supporting the hypothesis that neurodegeneration is primarily driven by inflammation [12, 14, 33, 35]. The presence of pMLKL along with MLKL tetramers, RIPK1 and MLKL in the insoluble protein fraction, as well as the interaction between RIP3 and MLKL in MS GM, indicates that necrosome formation was occurring in neurons, which could lead to destabilisation of the plasma membrane. Interestingly, pMLKL was also localized in the perinuclear and nuclear compartments, in agreement with previous studies demonstrating that MLKL can shuttle between the cytoplasm and the nucleus [60, 63]. This might suggest a mechanism through which neurons delay activation of cell death signaling, or it might indicate that pMLKL could be involved in destabilizing the nuclear membrane, as observed in other cell types [60, 63, 65].

We propose that cytokines, such as TNF, released in the meninges by infiltrating leukocytes, diffuse into the underlying GM to induce neurodegeneration via necroptotic mechanisms, both directly and indirectly. TNF and iNOS expression can be seen in microglia in the upper cortical layers of active MS GM lesions [35] and endogenous TNF and IFNγ expression can be induced in the rat cortex in response to chronically increased CSF levels of the same cytokines [21]. Our in vivo results confirm the relationship between meningeal inflammation and neurodegeneration, demonstrating that persistently increased TNF and INFγ in the CSF is sufficient to induce neuronal loss with the features of necroptosis after 28 days of chronic exposure. TNFR1 and pMLKL upregulation occurred in areas close to the midline where the inflammatory infiltrates were more extensive, which suggests that spatial association with the lymphoid aggregates induces more rapid neurodegeneration. While we found necroptosis activation in the surface cortical areas at 28 dpi, our previous studies demonstrated that neuronal loss also became significant in the deeper cortical layers by 56 dpi [21].

Whilst our results clearly indicate a role for increased TNF levels in neurodegeneration in MS, it is important to note that there are other triggers for RIPK3/MLKL dependent necroptotic cell death [15, 59, 65], including FasL, TRAIL, dsRNA and viral DNA, although their role in neuronal death has yet to be investigated. It is also not known whether necroptotic neuron death occurs in other CNS inflammatory conditions in which one might expect levels of TNF to be increased in the CNS. Although subpial demyelination appears to be specific to MS [9, 22], comparable studies on the role of cytokine-induced neuronal loss in the upper cortical layers in non-MS neuroinflammatory disorders with similar long disease duration have yet to be carried out.

In conclusion, we provide substantial evidence for TNF-mediated activation of necroptotic signaling in cortical neurons in MS, which we propose as a key mechanism that drives neurodegeneration and thereby contributes to the accumulation of clinical disability in the progressive phase of the disease. Evidence of necroptosis in other neurodegenerative diseases, such as Alzheimer’s disease [2, 25], amyotrophic lateral sclerosis [48] and Parkinson’s disease [19, 44], suggests that different pathologies may converge towards the same molecular mechanism to execute cell death. Definitive proof of the functional link between TNFR1 mediated necroptosis and neurodegeneration in MS-like pathology will require pharmacological intervention studies in our novel in vivo model, but when taken together with other evidence of necroptosis in MS [42], our data implies that targeting necroptosis could not only ameliorate TNF/TNFR1 induced neuronal cell death but also inhibit oligodendrocyte loss, thus having a dual action on demyelination and neurodegeneration. Indeed, brain penetrant RIPK1 inhibitors have recently entered clinical trials for use in AD and ALS [39] and hold great promise for progressive MS.

## Supplementary Information

Below is the link to the electronic supplementary material.

Supplementary file1 (DOCX 20 KB)

Supplementary file2 (DOCX 17 KB)

Supplementary file3. Supplemental Fig. 1 Neuropathological characterization of MS and control cases. **a** Sections were stained with anti-MOG antibody to quantify the extent of cortical GM demyelination in MS and control cases. Scale bar: 200 μm. Histogram shows the percentage of GM demyelination in each MS case (3 blocks analysed/case). **b** Immunostaining of snap-frozen sections with antibodies to CD3 (T cells) and CD20 (B cells) was used to quantify the number of cells within the perivascular cuffs in the subcortical areas. Scale bar: 20 μm. Histogram shows the total number of CD3+ plus CD20+ lymphocytes in eight perivascular spaces per MS case for each analysed case (3 blocks analysed/case). **c** Immunostaining of snap-frozen sections with antibodies to CD3 (T cells) and CD20 (B cells) was used to quantify the number of cells in the meningeal infiltrates. Scale bar: 20 μm. Histogram shows the number of CD3+ plus CD20+ lymphocytes in the meningeal space per tissue block for each individual MS case (3 blocks analysed/case). **d** Immunostaining of paraffin sections with anti-HLA-DR antibody was used to detect microglia/macrophages within the grey matter and to detect monocyte/macrophage infiltration in the subarachnoid space. Scale bar: 200 μm, 20 μm. Histogram shows the cortical HLA-DR+ cell density for each MS case (2 blocks analysed/case). **e** The graph shows the density of HLA-DR cells within all layers, layers I, II–III and V–VI in MS cases (*n* = 17) and controls (*n* = 10). **f** Immunostaining of paraffin sections with anti-HuC/D antibody was used to analyse the density of neurons in the cortical GM. Histogram shows the cortical HuC/D+ cell density for each MS case (2 blocks analysed/case). **g** The graph shows the density of HuC/D+ cells within total GM, layers II–III and V–VI in MS cases (*n* = 17) and controls (*n* = 10). Kruskal–Wallis followed by Dunn’s multiple comparisons test. Data are represented as mean ± SEM, *p < 0.05, **p < 0.01. (TIF 8156 KB)

Supplementary file4. Supplemental Fig. 2 Upregulation of TNFR1 and RIPK1 in MS GM. **a** Analysis of mRNA levels by QPCR for the TNFRI, TNFRII and RIPK1 genes in cortical GM in MS cases (*n* = 19) and controls (*n* = 10). **b** Analysis of the protein levels of CYLD in tissue lysates from the grey matter of MS (*n* = 25) and control (*n* = 9) cases. Band intensity values were normalized with β-actin. **c** Correlation analysis between RIPK1 protein levels and the degree of GM demyelination and the number of infiltrating lymphocytes in the subarachnoid space. **d** Double IHC staining of RIPK1 (brown) with GFAP, Iba-1 or Olig-2 (blue) in MS. Scale bars: 20 μm. Mann–Whitney test was used. Data are represented as mean ± SEM, **p < 0.01. (TIF 1398 KB)

Supplementary file5. Supplemental Fig. 3 Expression of cleaved caspase 3 in neurons in MS GM. Quantification of CC3+NeuN+ neuronal cell density (**a**) and the proportion of total neurons expressing CC3 (**b**) in layers II–III and V–VI in MS cases (*n* = 10) and controls (*n* = 5). Kruskal–Wallis followed by Dunn’s multiple comparisons test. Data are represented as mean ± SEM. (TIF 1121 KB)

Supplementary file6. Supplemental Fig. 4 Association between pMLKL cell density and myeloid cell activation. Correlation analysis showing a significant association between the number of cortical pMLKL+ cells and the density of activated HLA-DR+ microglia in the grey matter (**a**) and a non-significant relationship between cortical pMLKL+ cell density and HLA-DR+ macrophages in the meninges in progressive MS GM (*n *= 17). Correlation analysis by Spearman comparison. (TIF 756 KB)

Supplementary file7. Supplemental Fig. 5 Characterisation of inflammation, glial activation and neuronal loss in the rat model of cortical pathology. Immunofluorescent images illustrate the large increase in CD4+, CD8+ (**a**) and CD79a+ cells (**b**) in the sagittal sulcus of TNF-IFNγ vector injected animals (scale bar: 100 μm). Immunofluorescent images of Iba-1+ microglia/macrophages (**c**), GFAP+ astrocytes (**d**) and NeuN+ neurons (**e**) in the cortical layers in TNF/IFNγ vector injected, (*n* = 5) and naive rats (*n *= 4) and their quantification: Iba-1 cells (left), GFAP (middle) and NeuN (right) (scale bar: 200 μm). **f**, **g** Representative images of cortical GM from TNF+INFγ vector injected animals immunostained for MLKL (green) and NeuN (red), showing the nuclear localisation of MLKL, and pMLKL (green) and NeuN (red) showing the cytoplasmic localisation of pMLKL and the presence of aggregates (scale bar: 20 μm). One-way ANOVA with Bonferroni’s post-hoc correction. Data are represented as mean ± SEM, *p < 0.05, ***p < 0.001. (TIF 14831 KB)

Supplementary file8. Supplemental Fig. 6 Time course of LDH release and neurite degeneration by rat cortical neurons treated with TNF. **a** Representative images of cultured primary cortical neurons treated with TNF (100 ng/ml) or vehicle for 24 h and immunostained for NfH and NeuN. (Scale bar: 20 μm). **b** Quantification of NfH and NeuN fluorescence intensity after 24 h of treatment with TNF in the TNF treated group and vehicle (3 replicates per group). **c** Cytotoxicity of neurons treated with rat TNF (100 ng/ml), SMAC mimetic (2 μM) and Z-vad (10 μM) at different time points, assessed by measuring LDH release. **d** Representative immunofluorescence images for NfH in cortical neurons treated with TSZ and vehicle after 6 and 24 of treatment, showing beading in response to TSZ treatment after 6 h (Scale bar: 20 μm). **e** Western blotting analysis of MLKL expression of vehicle and TSZ treated primary cortical neurons after 3 h treatment. β-actin as loading control. **f** Representative immunofluorescence images showing pMLKL expression in NfH+ neurites in TSZ treated cortical neurons. (Scale bar: 20 μm). One-way ANOVA with Bonferroni’s post-hoc correction. Data are represented as mean ± SEM, *p < 0.05, **p < 0.01, ***p < 0.001. (TIF 1684 KB)

Supplementary file9. Supplemental Fig. 7 **a** Full blots of TNFR-I, FADD, RIPK1, Casp8, RIPK3/pRIPK3 and MLKL/pMLKL of samples from human post-mortem brains. Proteins were extracted with RIPA and the blots probed with the indicated antibodies. The levels of the protein of interest were normalized to ß-actin. **b** Full blots of RIPK1 and MLKL in samples from grey matter of human post-mortem brains. Proteins were extracted with UREA. The levels of the protein of interest were normalized to ß-actin. (TIF 2101 KB)
