# Supplementary material for: Neuron-specific activation of necroptosis signaling in multiple sclerosis cortical grey matter
Source: Acta Neuropathol. 2021 Feb 10;141(4):585–604. doi: 10.1007/s00401-021-02274-7 (PMC7952371; doi:10.1007/s00401-021-02274-7)
Supplement: Supplementary file 1 — Supplementary file1 (DOCX 20 KB) [file 401_2021_2274_MOESM1_ESM.docx]

Supplementary Table 1: Clinical and demographic data for MS and control brains

| Case ID | Gender | Age at death (yr) | PMD | Cause of Death | Age at onset (yr) | Disease duration (yr) | Time progressive (yr) | Age at progressive (yr) |
| --- | --- | --- | --- | --- | --- | --- | --- | --- |
| C72 | Male | 77 | 26 | Pneumonia, ischaemic bowel | | | | |
| PDC40 | Female | 61 | 15 | Ovarian cancer | | | | |
| PDC41 | Male | 66 | 23 | Carcinoma of the lung | | | | |
| PDC39 | Female | 50 | 28 | Metastatic renal cancer | | | | |
| PDC22 | Male | 65 | 12 | Squamous cell carcinoma | | | | |
| PDC8 | Female | 71 | 17 | Myocardial infarction | | | | |
| C25 | Male | 35 | 22 | Carcinoma of the tongue | | | | |
| C28 | Female | 60 | 13 | Ovarian cancer | | | | |
| PDC29 | Male | 82 | 48 | Metastatic liver and lung cancer | | | | |
| C48 | Male | 68 | 10 | Metastatic colon cancer | | | | |
| MS153 | Female | 50 | 12 | MS | 19 | 27 | 10 | 40 |
| MS317 | Female | 48 | 21 | Pneumonia MS | 20 | 26 | 20 | 30 |
| MS371 | Male | 40 | 27 | Pneumonia | 24 | 16 | 7 | 33 |
| MS377 | Female | 50 | 22 | Pneumonia MS | 27 | 23 | 5 | 45 |
| MS387 | Female | 42 | 13 | MS | 25 | 17 | 8 | 34 |
| MS389 | Female | 55 | 14 | MS, urinary sepsis | 28 | 27 | 12 | 43 |
| MS402 | Male | 46 | 12 | Pneumonia MS | 20 | 26 | 9 | 37 |
| MS403 | Female | 54 | 11 | MS | 28 | 26 | 15 | 39 |
| MS404 | Female | 55 | 17 | Septicaemia, shock and pneumonia | 21 | 34 | 20 | 35 |
| MS405 | Male | 62 | 12 | Septicaemia, MS, metastatic colon cancer | 37 | 25 | 20 | 43 |
| MS406 | Male | 62 | 23 | Aspiration pneumonia, MS | 18 | 43 | 20 | 42 |
| MS408 | Male | 39 | 21 | Pneumonia, sepsis | 29 | 11 | 5 | 34 |
| MS411 | Male | 61 | 24 | Pneumonia, MS | 32 | 29 | 15 | 46 |
| MS426 | Female | 48 | 21 | MS | 29 | 19 | 19 | 29 |
| MS430 | Female | 61 | 26 | Pneumonia | 21 | 40 | 22 | 39 |
| MS438 | Female | 53 | 17 | MS | 41 | 12 | 4 | 49 |
| MS445 | Female | 62 | 13 | Lower respiratory tract infection, MS | 24 | 38 | 13 | 49 |
| MS461 | Male | 43 | 13 | Pneumonia, MS | 20 | 21 | 8 | 35 |
| MS466 | Female | 65 | 25 | MS pneumonia,  lung & uterine cancer | 29 | 36 | 12 | 53 |
| MS473 | Female | 39 | 9 | Pneumonia, MS | 26 | 13 | 10 | 29 |
| MS491 | Female | 64 | 9 | Anaphylactic reaction | 39 | 28 | 11 | 53 |
| MS497 | Female | 60 | 26 | Aspiration pneumonia, MS | 31 | 29 | 12 | 46 |
| MS510 | Female | 38 | 19 | Pneumonia, MS | 16 | 22 | 15 | 23 |
| MS513 | Female | 51 | 17 | MS, respiratory failure | 33 | 18 | 15 | 36 |
| MS528 | Female | 45 | 17 | MS | 20 | 25 | 10 | 35 |
| MS530 | Male | 42 | 15 | MS | 21 | 21 | 15 | 27 |
| MS535 | Female | 65 | 12 | MS | 25 | 40 | 25 | 40 |
| MS549 | Male | 50 | 8 | MS | 21 | 29 | 20 | 30 |
| MS585 | Female | 53 | 27 | Pneumonia, MS | 26 | 27 | 6 | 47 |
| MS586 | Female | 58 | 27 | Respiratory failure | 20 | 38 | 10 | 48 |
| MS601 | Male | 70 | 11 | Pneumonia, MS | 34 | 36 | 17 | 53 |
