# Supplementary material for: Neuron-specific activation of necroptosis signaling in multiple sclerosis cortical grey matter
Source: Acta Neuropathol. 2021 Feb 10;141(4):585–604. doi: 10.1007/s00401-021-02274-7 (PMC7952371; doi:10.1007/s00401-021-02274-7)
Supplement: Supplementary file 2 — Supplementary file2 (DOCX 17 KB) [file 401_2021_2274_MOESM2_ESM.docx]

Supplementary Table 2: Antibody list

| **REAGENT or RESOURCE** | **SOURCE** | **IDENTIFIER** | **Application** |
| --- | --- | --- | --- |
| **Antibodies** |  |  |  |
| Mouse monoclonal anti-β-Actin (AC-15) antibody | Santa Cruz Biotechnology | Cat#sc-69879; RRID: AB_1119529 | WB (1:10000) |
| Mouse monoclonal anti-MLKL (32B) antibody | Santa Cruz Biotechnology | Cat#sc-ab187091 | IHC (1:200) |
| Rabbit polyclonal anti-phospho-human MLKL (Ser 358) antibody | Abcam | Cat# ab187091, RRID:AB_2619685 | WB (1:2000); IHC (1:100) |
| Rabbit polyclonal anti- MLKL antibody | Abcam | ab183770 | WB (1:2000) |
| Rabbit polyclonal anti-phospho-human RIP3 (Ser 227) antibody | Abcam | Cat# ab209384, RRID:AB_2714035 | IHC (1:500) |
| Mouse monoclonal anti-RIP (7H10) antibody | Abcam | Cat# ab72139, RRID:AB_2178115 | WB (1:2000); IHC (1:200) |
| Rabbit monoclonal anti-CD20 antibody | Abcam | Cat# ab78237, RRID:AB_1640323 | IHC (1:200) |
| Rabbit monoclonal anti-TNF receptor II antibody | Abcam | ab109322 | WB (1:1000) |
| Rabbit monoclonal anti-Olig-2 antibody | Abcam | Cat# ab109186, RRID:AB_10861310 | IHC (1:200) |
| Rabbit monoclonal anti-NeuN antibody | Abcam | Cat# ab177487, RRID:AB_2532109 | IHC (1:1000) |
| Rabbit polyclonal anti-FADD antibody | Cell Signaling Technology | Cat# 2782, RRID:AB_2100484 | WB (1:1000) |
| Rabbit monoclonal anti-CYLD (D1A10) antibody | Cell Signaling Technology | Cat# 8462, RRID:AB_10949157 | WB (1:2000) |
| Rabbit polyclonal anti-Cleaved caspase 3 (Asp175) antibody | Cell Signaling Technology | Cat# 9661, RRID:AB_2341188 | IHC (1:400) |
| Mouse monoclonal anti-caspase-8 (IC12) antibody | Cell Signaling Technology | Cat# 9746, RRID:AB_2275120 | WB (1:1000) |
| Mouse monoclonal anti-human RIPK3 antibody | R and D Systems | Cat# MAB7604, RRID:AB_2619684 | IP |
| Mouse monoclonal anti-human TNFRI antibody | R and D Systems | Cat# MAB225, RRID:AB_2204150 | WB (1:1000); IHC (1:200) |
| Mouse monoclonal anti-human phospho-MLKL (T357) antibody | R and D Systems | MAB9187 | IHC (1:200) |
| Mouse monoclonal anti-NeuN antibody | Millipore | at# MAB377, RRID:AB_2298772 | IHC (1:500) |
| Mouse polyclonal anti-NeuN antibody | Millipore | Cat# ABN91, RRID:AB_11205760 | IHC (1:1000) |
| Rabbit polyclonal anti-Iba-1 antibody | Wako | Cat# 019-19741, RRID:AB_839504 | IHC (1:500) |
| Mouse monoclonal anti-human HLA-DP,DQ,DR antibody | Aligent | Cat# M0775, RRID:AB_2313661 | IHC (1:500) |
| Rabbit polyclonal anti-GFAP antibody | Aligent | Cat# Z0334, RRID:AB_10013382 | IHC (1:500) |
| Rabbit polyclonal anti- CD3 antibody | Aligent | Cat# A0452, RRID:AB_2335677 | IHC (1:200) |
| Mouse monoclonal anti-GFAP antibody | Sigma-Aldrich | Cat# G3893, RRID:AB_47701 | IHC (1:500) |
| Horse Anti-Mouse IgG Antibody (H+L), Biotinylated | Vector laboratories | Cat# BA-2000, RRID:AB_2313581 | IHC (1:500) |
| Horse Anti-Rabbit IgG Antibody (H+L), Biotinylated | Vector laboratories | Cat# BA-1100, RRID:AB_2336201 | IHC (1:500) |
| ImmPACT® DAB Substrate, Peroxidase (HRP) | Vector Laboratories | Cat# SK-4105, RRID:AB_2336520 | |
| Vector® Blue Substrate Kit, Alkaline Phosphatase (AP) | Vector Laboratories | Cat# SK-5300, RRID:AB_2336837 | |
| VECTASTAIN® ABC-AP Kit, Alkaline Phosphatase | Vector Laboratories | AK-500 |  |
| ImmPRESS® HRP Horse Anti-Rabbit IgG Polymer Detection Kit, Peroxidase | Vector Laboratories | Cat# MP-7401, RRID:AB_2336529 | |
| ImmPRESS® HRP Horse Anti-Mouse IgG Polymer Detection Kit, Peroxidase | Vector Laboratories | Cat# MP-7422, RRID:AB_2336527 | |
| ImmPRESS® HRP Horse Anti-Mouse IgG, Rat adsorbed Polymer Detection Kit, Peroxidase | Vector Laboratories | MP-7403 |  |
| Goat anti-Rabbit IgG (H+L) Highly Cross-Adsorbed Secondary Antibody, Alexa Fluor Plus 488 | ThermoFisher | Cat# A32731, RRID:AB_2633280 | IHC (1:1000) |
| Goat Anti-Mouse IgG1 Antibody, Alexa Fluor 488 Conjugated | ThermoFisher | Cat# A-21121, RRID:AB_2535764 | IHC (1:1000) |
| Goat Anti-Mouse IgG2a Antibody, Alexa Fluor 488 Conjugated | ThermoFisher | Cat# A-21131, RRID:AB_141618 | IHC (1:1000) |
| Goat anti-Chicken IgY (H+L) Cross-Adsorbed Secondary Antibody, Alexa Fluor Plus 647 | ThermoFisher | Cat# A32933, RRID:AB_2762845 | IHC (1:1000) |
| Goat anti-Mouse IgG1 Cross-Adsorbed Secondary Antibody, Alexa Fluor 555 | ThermoFisher | A-21127, RRID:AB_141596 | IHC (1:1000) |
| Goat anti-Mouse IgG (H+L) Cross-Adsorbed Secondary Antibody, Alexa Fluor 555 | ThermoFisher | A-21422, RRID:AB_141822 | IHC (1:1000) |
| Peroxidase AffiniPure Goat Anti-Rabbit IgG | Jackson ImmunoReseach | Cat#111-035-144,RRID:AB_2307391 | WB (1:10000) |
| Peroxidase AffiniPure Goat Anti-Mouse IgG | Jackson ImmunoReseach | Cat#115-035-003,RRID: AB_10015289 | WB (1:1000) |
