# Supplementary figures and images for: Neuron-specific activation of necroptosis signaling in multiple sclerosis cortical grey matter
Source: Acta Neuropathol. 2021 Feb 10;141(4):585–604. doi: 10.1007/s00401-021-02274-7 (PMC7952371; doi:10.1007/s00401-021-02274-7)

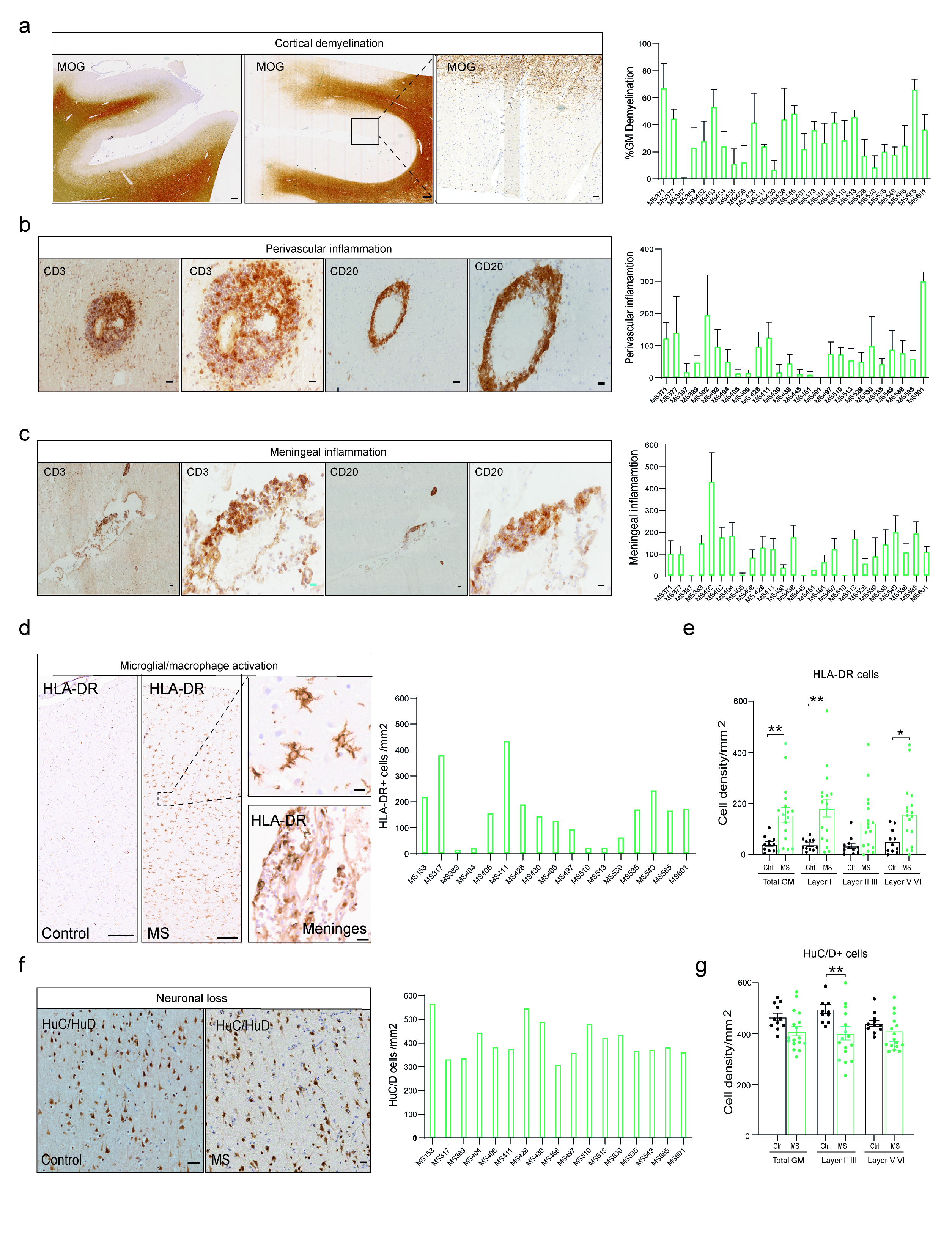

Supplement: Supplementary file 3 — Supplementary file3. Supplemental Fig. 1 Neuropathological characterization of MS and control cases. a Sections were stained with anti-MOG antibody to quantify the extent of cortical GM demyelination in MS and control cases. Scale bar: 200 μm. Histogram shows the percentage of GM demyelination in each MS case (3 blocks analysed/case). b Immunostaining of snap-frozen sections with antibodies to CD3 (T cells) and CD20 (B cells) was used to quantify the number of cells within the perivascular cuffs in the subcortical areas. Scale bar: 20 μm. Histogram shows the total number of CD3+ plus CD20+ lymphocytes in eight perivascular spaces per MS case for each analysed case (3 blocks analysed/case). c Immunostaining of snap-frozen sections with antibodies to CD3 (T cells) and CD20 (B cells) was used to quantify the number of cells in the meningeal infiltrates. Scale bar: 20 μm. Histogram shows the number of CD3+ plus CD20+ lymphocytes in the meningeal space per tissue block for each individual MS case (3 blocks analysed/case). d Immunostaining of paraffin sections with anti-HLA-DR antibody was used to detect microglia/macrophages within the grey matter and to detect monocyte/macrophage infiltration in the subarachnoid space. Scale bar: 200 μm, 20 μm. Histogram shows the cortical HLA-DR+ cell density for each MS case (2 blocks analysed/case). e The graph shows the density of HLA-DR cells within all layers, layers I, II–III and V–VI in MS cases (n = 17) and controls (n = 10). f Immunostaining of paraffin sections with anti-HuC/D antibody was used to analyse the density of neurons in the cortical GM. Histogram shows the cortical HuC/D+ cell density for each MS case (2 blocks analysed/case). g The graph shows the density of HuC/D+ cells within total GM, layers II–III and V–VI in MS cases (n = 17) and controls (n = 10). Kruskal–Wallis followed by Dunn’s multiple comparisons test. Data are represented as mean ± SEM, *p < 0.05, **p < 0.01. (TIF 8156 KB) [file 401_2021_2274_MOESM3_ESM.tif]

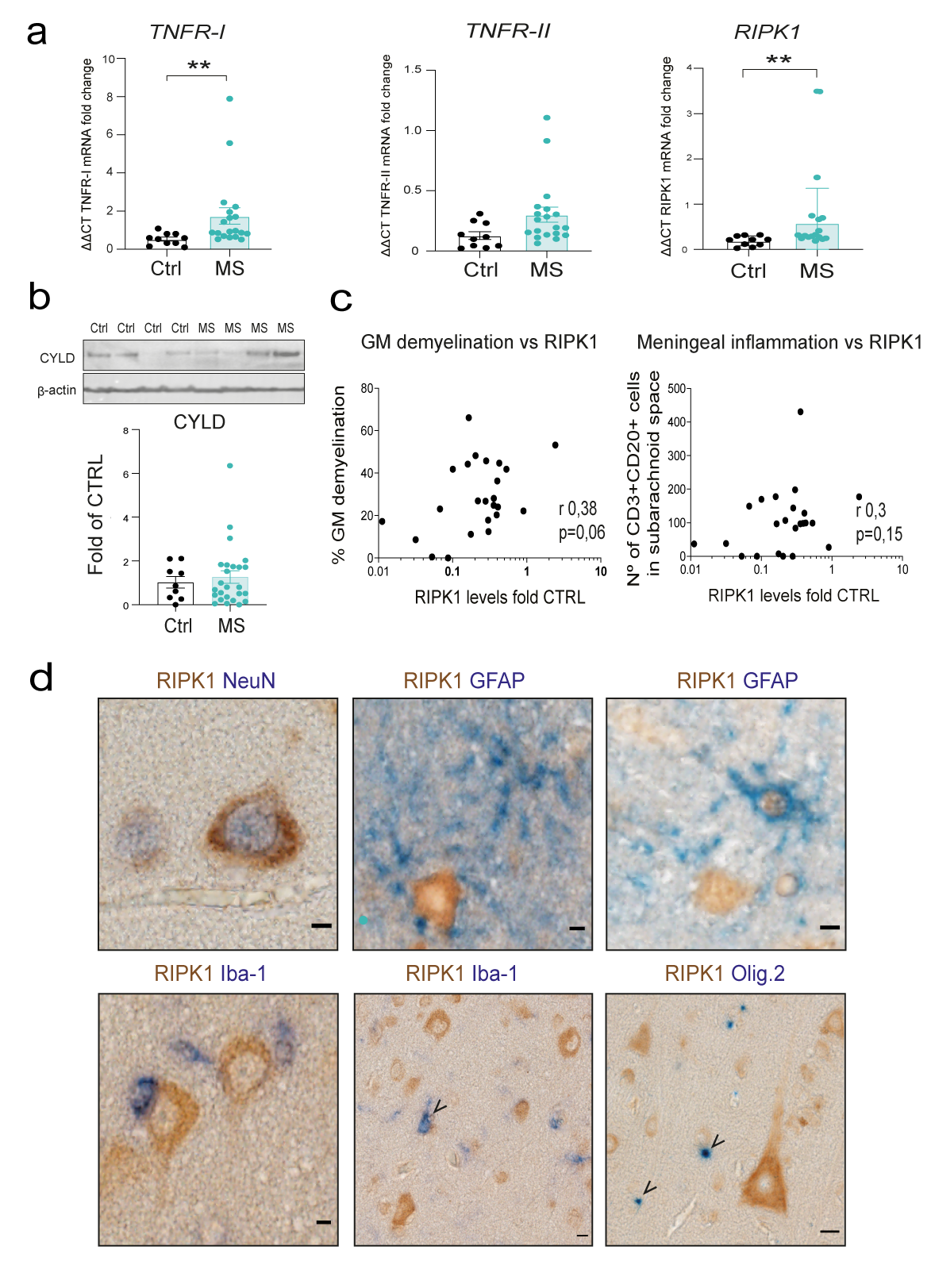

Supplement: Supplementary file 4 — Supplementary file4. Supplemental Fig. 2 Upregulation of TNFR1 and RIPK1 in MS GM. a Analysis of mRNA levels by QPCR for the TNFRI, TNFRII and RIPK1 genes in cortical GM in MS cases (n = 19) and controls (n = 10). b Analysis of the protein levels of CYLD in tissue lysates from the grey matter of MS (n = 25) and control (n = 9) cases. Band intensity values were normalized with β-actin. c Correlation analysis between RIPK1 protein levels and the degree of GM demyelination and the number of infiltrating lymphocytes in the subarachnoid space. d Double IHC staining of RIPK1 (brown) with GFAP, Iba-1 or Olig-2 (blue) in MS. Scale bars: 20 μm. Mann–Whitney test was used. Data are represented as mean ± SEM, **p < 0.01. (TIF 1398 KB) [file 401_2021_2274_MOESM4_ESM.tif]

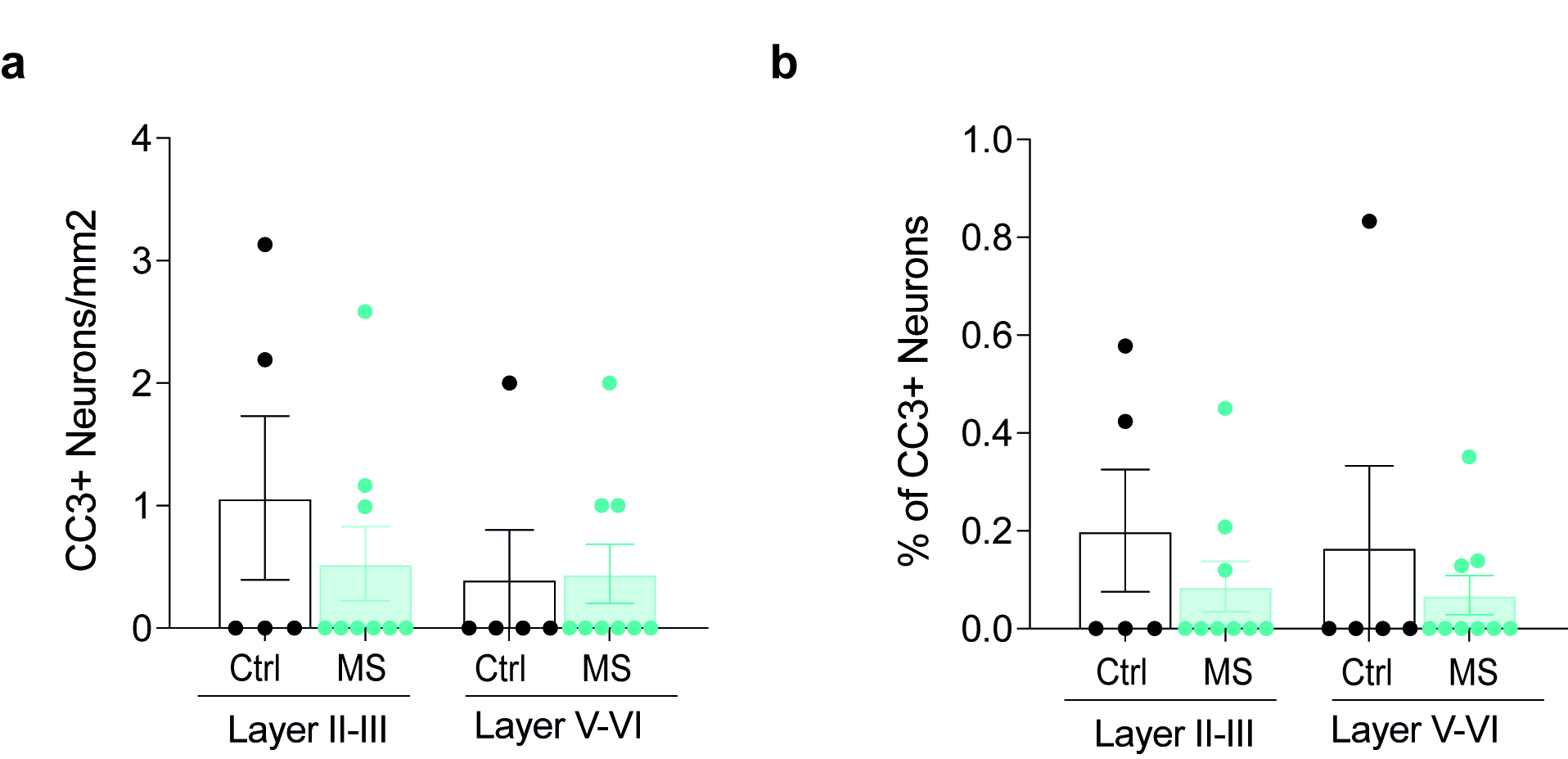

Supplement: Supplementary file 5 — Supplementary file5. Supplemental Fig. 3 Expression of cleaved caspase 3 in neurons in MS GM. Quantification of CC3+NeuN+ neuronal cell density (a) and the proportion of total neurons expressing CC3 (b) in layers II–III and V–VI in MS cases (n = 10) and controls (n = 5). Kruskal–Wallis followed by Dunn’s multiple comparisons test. Data are represented as mean ± SEM. (TIF 1121 KB) [file 401_2021_2274_MOESM5_ESM.tif]

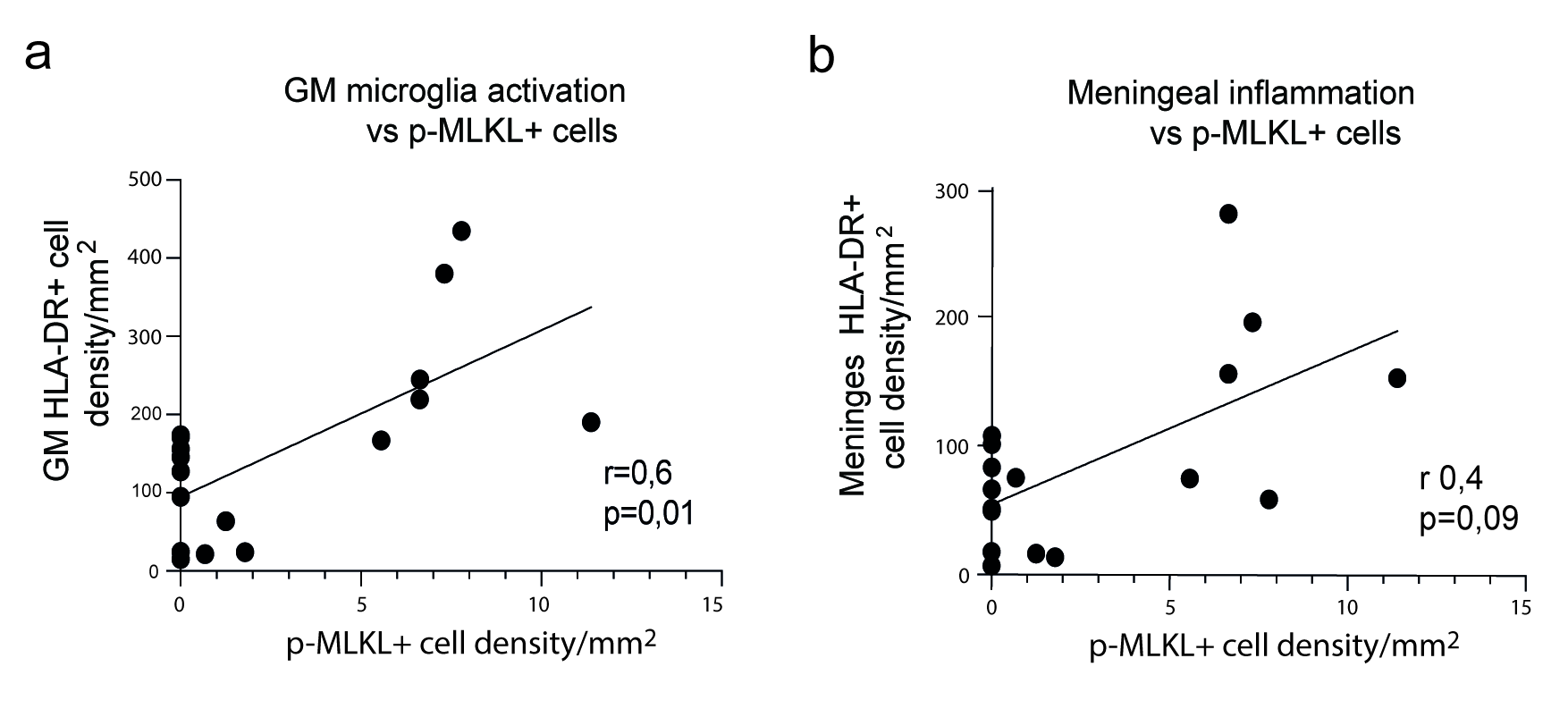

Supplement: Supplementary file 6 — Supplementary file6. Supplemental Fig. 4 Association between pMLKL cell density and myeloid cell activation. Correlation analysis showing a significant association between the number of cortical pMLKL+ cells and the density of activated HLA-DR+ microglia in the grey matter (a) and a non-significant relationship between cortical pMLKL+ cell density and HLA-DR+ macrophages in the meninges in progressive MS GM (n = 17). Correlation analysis by Spearman comparison. (TIF 756 KB) [file 401_2021_2274_MOESM6_ESM.tif]

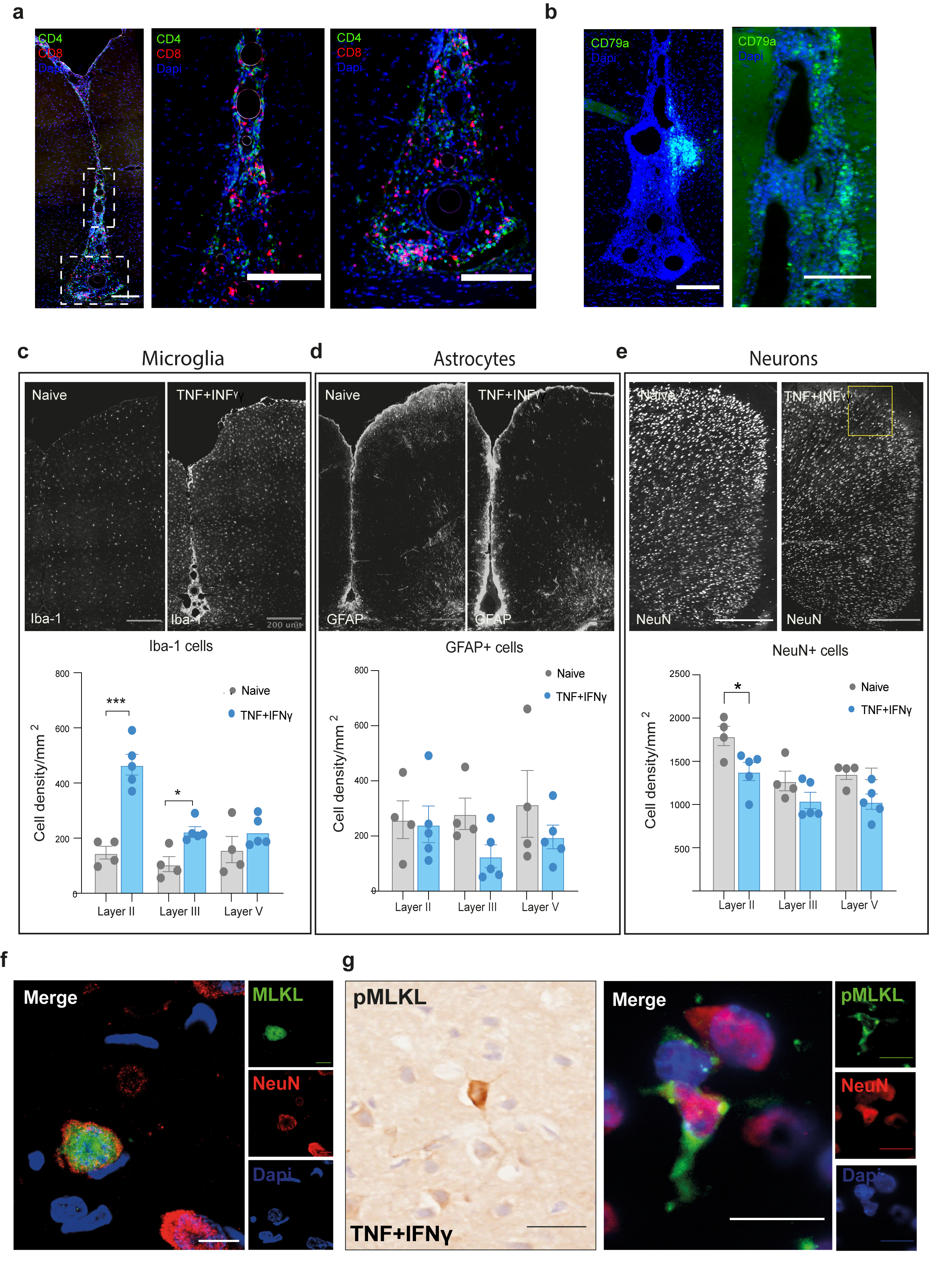

Supplement: Supplementary file 7 — Supplementary file7. Supplemental Fig. 5 Characterisation of inflammation, glial activation and neuronal loss in the rat model of cortical pathology. Immunofluorescent images illustrate the large increase in CD4+, CD8+ (a) and CD79a+ cells (b) in the sagittal sulcus of TNF-IFNγ vector injected animals (scale bar: 100 μm). Immunofluorescent images of Iba-1+ microglia/macrophages (c), GFAP+ astrocytes (d) and NeuN+ neurons (e) in the cortical layers in TNF/IFNγ vector injected, (n = 5) and naive rats (n = 4) and their quantification: Iba-1 cells (left), GFAP (middle) and NeuN (right) (scale bar: 200 μm). f, g Representative images of cortical GM from TNF+INFγ vector injected animals immunostained for MLKL (green) and NeuN (red), showing the nuclear localisation of MLKL, and pMLKL (green) and NeuN (red) showing the cytoplasmic localisation of pMLKL and the presence of aggregates (scale bar: 20 μm). One-way ANOVA with Bonferroni’s post-hoc correction. Data are represented as mean ± SEM, *p < 0.05, ***p < 0.001. (TIF 14831 KB) [file 401_2021_2274_MOESM7_ESM.tif]

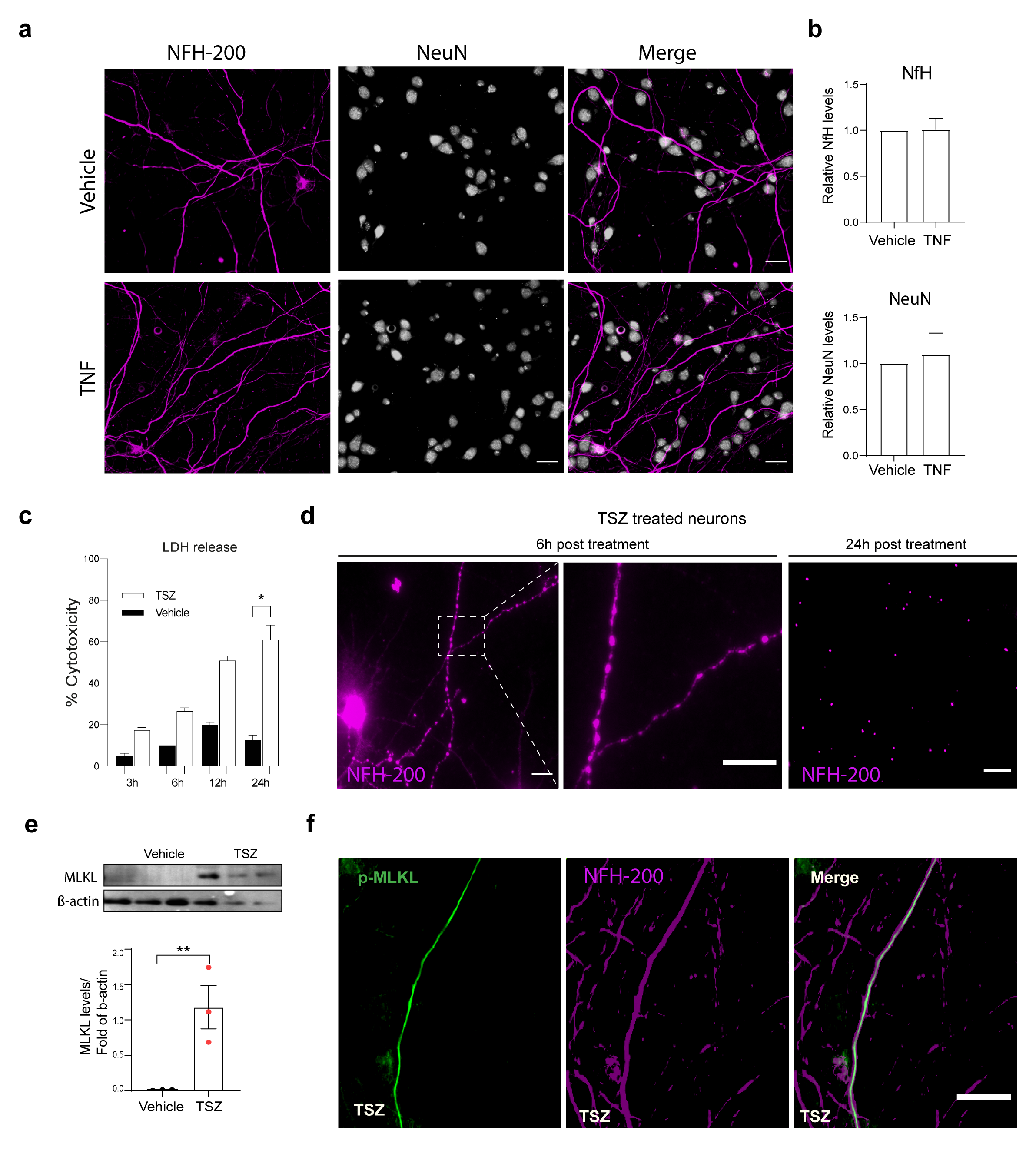

Supplement: Supplementary file 8 — Supplementary file8. Supplemental Fig. 6 Time course of LDH release and neurite degeneration by rat cortical neurons treated with TNF. a Representative images of cultured primary cortical neurons treated with TNF (100 ng/ml) or vehicle for 24 h and immunostained for NfH and NeuN. (Scale bar: 20 μm). b Quantification of NfH and NeuN fluorescence intensity after 24 h of treatment with TNF in the TNF treated group and vehicle (3 replicates per group). c Cytotoxicity of neurons treated with rat TNF (100 ng/ml), SMAC mimetic (2 μM) and Z-vad (10 μM) at different time points, assessed by measuring LDH release. d Representative immunofluorescence images for NfH in cortical neurons treated with TSZ and vehicle after 6 and 24 of treatment, showing beading in response to TSZ treatment after 6 h (Scale bar: 20 μm). e Western blotting analysis of MLKL expression of vehicle and TSZ treated primary cortical neurons after 3 h treatment. β-actin as loading control. f Representative immunofluorescence images showing pMLKL expression in NfH+ neurites in TSZ treated cortical neurons. (Scale bar: 20 μm). One-way ANOVA with Bonferroni’s post-hoc correction. Data are represented as mean ± SEM, *p < 0.05, **p < 0.01, ***p < 0.001. (TIF 1684 KB) [file 401_2021_2274_MOESM8_ESM.tif]

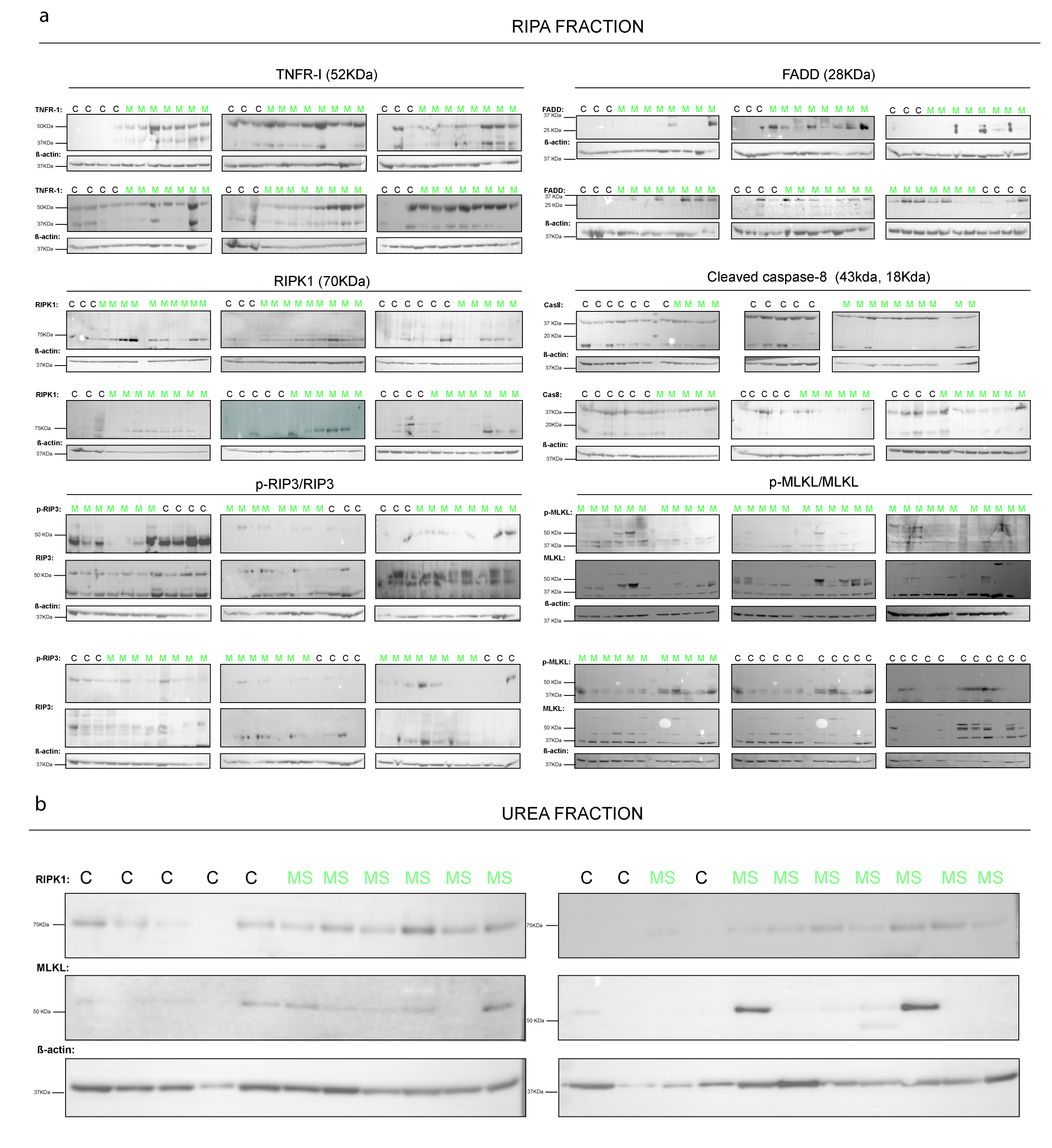

Supplement: Supplementary file 9 — Supplementary file9. Supplemental Fig. 7 a Full blots of TNFR-I, FADD, RIPK1, Casp8, RIPK3/pRIPK3 and MLKL/pMLKL of samples from human post-mortem brains. Proteins were extracted with RIPA and the blots probed with the indicated antibodies. The levels of the protein of interest were normalized to ß-actin. b Full blots of RIPK1 and MLKL in samples from grey matter of human post-mortem brains. Proteins were extracted with UREA. The levels of the protein of interest were normalized to ß-actin. (TIF 2101 KB) [file 401_2021_2274_MOESM9_ESM.tif]
